# The transcription factors *VaERF16* and *VaMYB306* interact to enhance resistance of grapevine to *Botrytis cinerea* infection

**DOI:** 10.1111/mpp.13223

**Published:** 2022-07-12

**Authors:** Yanxun Zhu, Xiuming Zhang, Qihan Zhang, Shengyue Chai, Wuchen Yin, Min Gao, Zhi Li, Xiping Wang

**Affiliations:** ^1^ State Key Laboratory of Crop Stress Biology in Arid Areas College of Horticulture Northwest A&F University Yangling China; ^2^ 12469 Key Laboratory of Horticultural Plant Biology and Germplasm Innovation in Northwest China Ministry of Agriculture Northwest A&F University Yangling China

**Keywords:** *Botrytis cinerea*, disease resistance, ERF transcription factor, grapevine, MYB transcription factor

## Abstract

*Botrytis cinerea* is a fungus that infects cultivated grape (*Vitis vinifera*); the identification and characterization of resistance mechanisms in the host is of great importance for the grape industry. Here, we report that a transcription factor in the ethylene‐responsive factor (ERF) family (*VaERF16*) from Chinese wild grape (*Vitis amurensis* ‘Shuang You’) is expressed during *B*. *cinerea* infection and in response to treatments with the hormones ethylene and methyl jasmonate. Heterologous overexpression of *VaERF16* in *Arabidopsis thaliana* substantially enhanced resistance to *B*. *cinerea* and the bacterium *Pseudomonas syringae* DC3000 via the salicylic acid and jasmonate/ethylene signalling pathways. Yeast two‐hybrid, bimolecular fluorescence complementation, and co‐immunoprecipitation assays indicated that VaERF16 interacts with the MYB family transcription factor VaMYB306. Overexpression of *VaERF16* or *VaMYB306* in grape leaves increased resistance to *B*. *cinerea* and caused an up‐regulation of the defence‐related gene *PDF1.2*, which encodes a defensin‐like protein. Conversely, silencing of either gene resulted in increased susceptibility to *B. cinerea*. Yeast one‐hybrid and dual‐luciferase assays indicated that VaERF16 increased the transcript levels of *VaPDF1.2* by binding directly to the GCC box in its promoter. Notably, VaMYB306 alone did not bind to the *VaPDF1.2* promoter, but the VaERF16–VaMYB306 transcriptional complex resulted in higher transcript levels of *VaPDF1.2*, suggesting that the proteins function through their mutual interaction. Elucidation of this regulatory module may be of value in enhancing resistance of grapevine to *B. cinerea* infection.

## Graphical abstract

The ethylene‐responsive factor VaERF16 from *Vitis amurensis* ‘Shuang You’ enhances resistance to *Botrytis cinerea* by interacting with VaMYB306. The data provide new insights into the functions of ERF genes in response to pathogen inoculation.

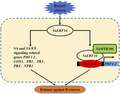

## 1 INTRODUCTION

Grapevine (*Vitis*) is an economically important fruit crop in many parts of the world, but in parallel with the expansion of areas used for grape cultivation, biotic stresses are increasingly challenging the grape industry. A particularly notable problem is infection with the fungus *Botrytis cinerea*, which causes one of the most harmful diseases that affect grape production. Yield loss caused by *B*. *cinerea* can reach more than 60% (Dean et al., 2012; Martínez‐Romero et al., 2007; Saito et al., 2019). However, a range of disease‐resistant wild grapevine genotypes from China have been identified, and the resistance of many accessions to *B*. *cinerea* has been evaluated using field and in vitro inoculation assays in previous studies. The results indicated that the fruits from 41 varieties and leaves from 81 varieties showed high resistance to *B. cinerea*. Slow spore development, reduced production of reactive oxygen species (ROS), higher antioxidant function, and high transcript levels of defence‐related genes were found in grape varieties with high resistance to *B. cinerea* (Rahman et al., 2019). Another study observed that *Vitis amurensis* ‘Shuang You’, ‘Tonghua‐3’, and ‘Taishan‐11’, *Vitis yenshanensis* ‘Yanshan‐1’, *Vitis* sp. (Qinling grape) ‘Pingli‐5’, and *Vitis adstricta* ‘Taishan‐2’ are highly resistant to *B*. *cinerea*. Hyphae grew more slowly on the leaves of highly resistant grape varieties and the area of disease spots was much smaller (Wan et al., 2015). Thus, the study of gene functions and disease resistance mechanisms and associated transcriptional regulatory networks in wild grapevine genotypes has great potential for grape improvement.

Members of the MYB transcription factor (TF) family contain one or more conserved MYB DNA‐binding domains, each consisting of 51–53 amino acid residues (Dubos et al., 2010). Based on the different numbers of MYB domains, the MYB TFs can be divided into four main families: 4R‐MYB, R1R2R3‐MYB, R2R3‐MYB, and 1R‐MYB (Stracke et al., 2001). The R2R3‐MYB TFs, which contains two repeated MYB domains, typically represent the largest group within the MYB TFs in plants. In recent years, the roles of R2R3‐MYB TFs in regulating responses to biotic stress in plants have been studied (Yu et al., 2019). For example, heterologous overexpression of *MdMYB30* from apple (*Malus domestica*) was shown to cause a hypersensitive reaction response and to enhance resistance to different bacterial pathogens in *Arabidopsis thaliana* (Zhang et al., 2019), and *AtMYB96* from *A. thaliana* was reported to be important for immune responses to the bacterium *Pseudomonas syringae* by regulating defence‐related genes in the salicylic acid (SA) signalling pathway (Seo & Park, 2010). In contrast, overexpression of *AtMYB46* in *A. thaliana* was found to decrease resistance to *B*. *cinerea* (Ramírez et al., 2011), so the functions and actions of MYB TFs in disease resistance are complex and not readily predictable. Notably, the roles of MYB TFs in responses of grape to *B*. *cinerea* have not been resolved.

The APETALA2/ethylene‐responsive factor (AP2/ERF) superfamily of TFs is also involved in regulating plant responses to *B. cinerea*, as well as growth and development (Li et al., 2015; Licausi et al., 2013). According to the different numbers of conserved AP2 domains, the AP2/ERF superfamily can be divided into three families: AP2, RAV, and ERF. Among them, members of the ERF family contain a single conserved AP2 domain (Nakano et al., 2006); the ERF family is the largest subfamily of the AP2/ERF superfamily (Gutterson & Reuber, 2004; Kizis et al., 2001). ERF proteins can specifically bind to GCC boxes (AGCCGCC), which are found in the promoters of biotic stress‐related genes (Fujimoto et al., 2000; Oñate‐Sánchez & Singh, 2002). The roles of ERF genes in response to *B*. *cinerea* challenge have mainly been studied in *A. thaliana*. For example, *ERF1* was shown to be expressed in response to different necrotrophic pathogens, such as *B*. *cinerea*, and after infection with *B*. *cinerea*, the jasmonic acid (JA)/ethylene (ET) signalling pathways were shown to be triggered, thereby transcriptionally activating *ERF1* and defence‐related genes. Silencing of the AP2/ERF gene *ORA59* in rice decreased resistance to *B*. *cinerea*, and it has also been shown that *ERF1* and *ORA59* are co‐activated by the JA/ET signalling pathway after inoculation with *B. cinerea* (Lorenzo et al., 2003; Pré et al., 2008). *RAP2.2* is a group VII ERF gene that is known to be a regulator of the ET signalling pathway in response to *B*. *cinerea* (Zhao et al., 2012). RAP2.2 has been found to interact with phytochrome and flowering time 1 (PFT1) as part of the JA signal transduction pathway and in response to *B*. *cinerea*, probably in the form of a complex (Kidd et al., 2009; Ou et al., 2011).

Recent studies have revealed that grape ERF genes also play key roles in *B*. *cinerea* resistance. For example, heterologous overexpression of *VqERF072*, *VqERF112*, and *VqERF114* from *Vitis quinquangularis* and *VaERF20* from *V. amurensis* in *A. thaliana* enhanced resistance to *B*. *cinerea* via the JA/ET signalling pathway and increased the transcript levels of defence‐related genes (Wang et al., 2018, 2020). In another study, the expression profiles of ERF genes at different time points after inoculation with *B*. *cinerea* in *B. cinerea*‐susceptible *Vitis vinifera* 'Red Globe' and the Chinese wild‐growing *V. amurensis* ‘Shuang You’, which is resistant to *B. cinerea* (Wan et al., 2015), indicated that most were up‐regulated and suggested networks of genes that contribute to immunity (Zhu et al., 2019). A previous analysis also showed that the transcript levels of *ERF16* from *V. vinifera* are induced by *B*. *cinerea* and that many stress‐responsive elements are located in the *ERF16* promoter (Zhu et al., 2019).

Here, we describe the characterization of a defence‐related regulatory module in *V*. *amurensis* ‘Shuang You’ involving ERF16 (encoded by *VaERF16*) and a MYB family TF (encoded by *VaMYB306*). Our results provide insight into resistance against a fungus that is increasingly problematic for grape cultivation and can be used to develop strategies to generate *B*. *cinerea*‐resistant grape cultivars.

## 2 RESULTS

### 2.1 Sequence analysis and expression patterns of *VaERF16*

**FIGURE 1:**
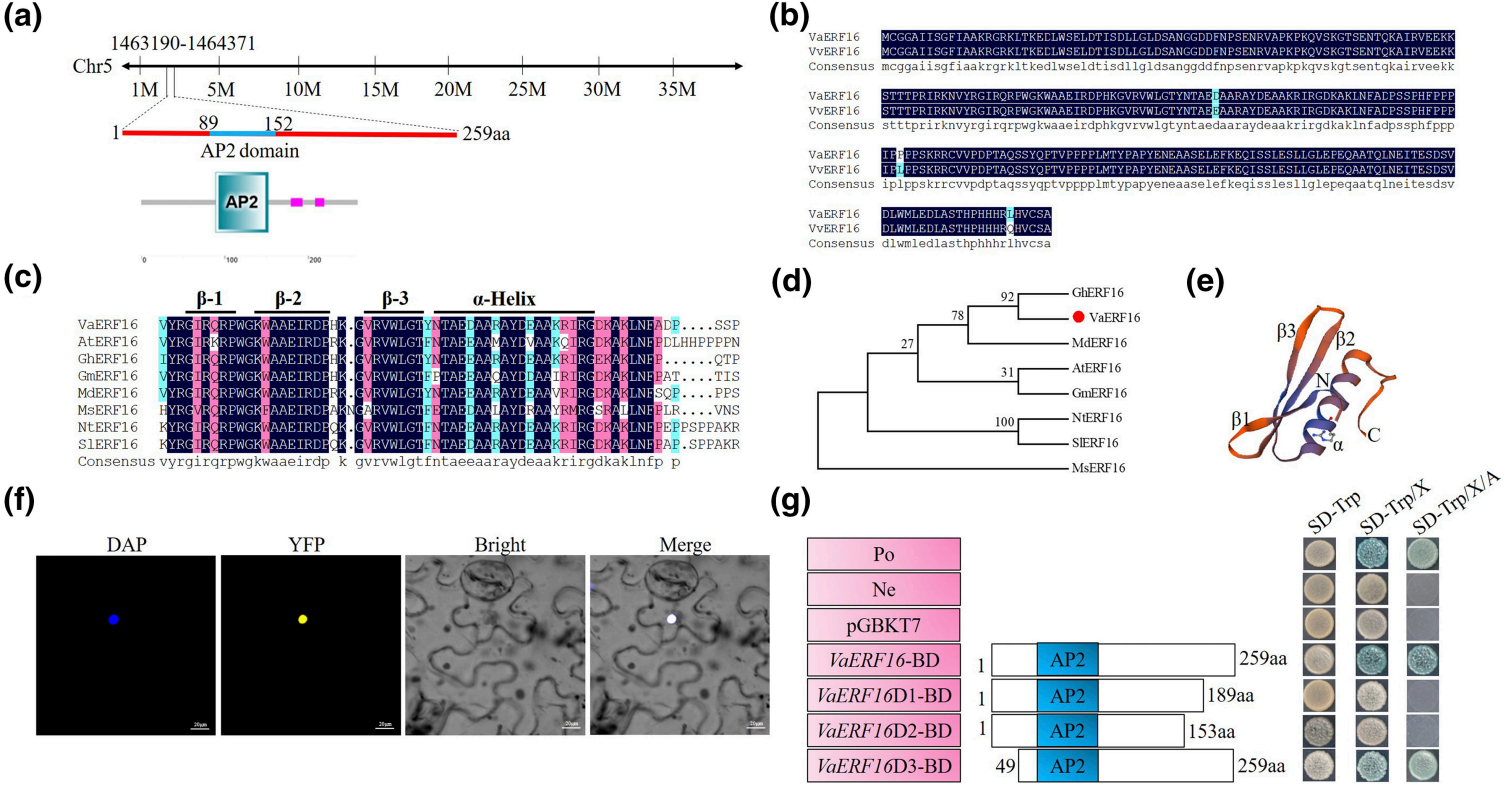
Sequence analysis of *VaERF16* isolated from *Vitis amurensis* ‘Shuang You’. (a) Chromosomal location of *VaERF16*. *VaERF16* is located on chromosome 5, from position 1,463,190 to 1,464,371. The AP2 domain (amino acids 89 to 152) is indicated with a blue line. (b) Sequence alignment of VaERF16 and VvERF16. Differences are highlighted in blue. (c) Multiple sequence alignment of VaERF16 and its homologues. A single α‐helix and three β‐sheets are marked with black lines. The sequences are from the following proteins: AtERF16 (*Arabidopsis thaliana*, AAC49769.1), GhERF16 (*Gossypium hirsutum*, AAX68525.1), GmERF16 (*Glycine max*, NP_001243393.1), MdERF16 (*Malus domestica*, NP_001315660.1), MsERF16 (*Medicago sativa*, AEQ64867.1), NtERF16 (*Nicotiana tabacum*, XP_016447100), and SlERF16 (*Solanum lycopersicum*, NP_001266125.1). (d) Phylogenetic analysis of VaERF16 (indicated with a red circle). (e) Predicted three‐dimensional structure of VaERF16. (f) Subcellular localization of VaERF16 in tobacco leaves. 4′,6‐Diamidino‐2‐phenylindole (DAP) staining was applied to stain the nucleus. Yellow fluorescent protein (YFP) signals were detected with a laser confocal microscope. Scale bar = 20 μm. (g) *VaERF16* transactivation assay in yeast. Co‐transformation of AD/T with BD/p53 or BD/Lam into yeast cells was used as positive (Po) and negative controls (Ne), respectively. Abbreviations: SD−Trp/X, SD−Trp/X‐α‐Gal; SD−Trp/X/A, SD−Trp/X‐α‐Gal/aureobasidin A

We previously identified 113 grape ERF family genes through the hidden Markov model (HMM) profile of the AP2 domain (PF00847) and examined the transcript levels of ERF genes from *V. vinifera* as well as *V. amurensis* in response to *B*. *cinerea* infection. *ERF16* (GenBank accession no. CBI22960.3) contains one conserved AP2 domain and its expression is induced by *B. cinerea* infection (Zhu et al., 2019). To further understand the potential function of *ERF16* in grape pathogen resistance, we cloned and sequenced the full‐length *VaERF16* cDNA sequence derived from the leaves of *V. amurensis* ‘Shuang You’. *VaERF16* was found to comprise a 777‐bp open reading frame encoding a 259‐amino‐acid protein with a predicted molecular weight of 28.72 kDa. The Grape Genome Browser (https://www.genoscope.cns.fr/externe/GenomeBrowser/Vitis/) indicated that *VaERF16* is located on chromosome 5 (Figure 1a). VaERF16, which has a conserved AP2 domain (amino acid residues 89–152), is predicted to differ by only three amino acids from *V. vinifera* VvERF16 (Figure 1b). The ERF family has been identified in various plant species such as *Arabidopsis* (Nakano et al., 2006), tobacco (Gao et al., 2020), soybean (Zhang et al., 2008), apple (Girardi et al., 2013), tomato (Yang et al., 2021), cotton (Liu & Zhang, 2017), and alfalfa (Jin et al., 2019). Thus, we selected seven homologous genes of *VaERF16* from these plant species for sequence alignment. The results showed that *VaERF16* has high sequence similarity to cotton *GhERF16* (GenBank accession no. AAX68525.1) (Figure 1c,d). A three‐dimensional structure prediction of VaERF16 using the SWISS‐MODEL database (https://swissmodel.expasy.org/) revealed a long C‐terminal α‐helix together with a three‐stranded antiparallel β‐sheet (from β1 to β3), which is similar to the previously reported *A. thaliana* AP2 domain structure (Nakano et al., 2006), suggesting a high degree of evolutionary conservation (Figure 1e). The subcellular localization of VaERF16 was investigated by heterologous expression of a VaERF16‐yellow fluorescent protein (YFP) fusion protein in *Nicotiana benthamiana*. The resulting fluorescent signal co‐localized with that of the nuclear marker 4′,6‐diamidino‐2‐phenylindole (DAPI), indicating that VaERF16 is a nuclear protein (Figure 1f). In addition, a yeast two‐hybrid (Y2H) assay showed that VaERF16 has transcriptional activation activity, because the yeast strain expressing the VaERF16‐BD protein grew well and showed activation of the *GAL4* reporter gene when grown on SD/−Trp/X‐α‐Gal/aureobasidin A (AbA) medium (Figure 1g). To avoid autoactivation in subsequent studies, we made truncated constructs and found that both a C‐terminal 70‐amino‐acid deletion (*VaERF16*D1‐BD) and a C‐terminal 106‐amino‐acid deletion (*VaERF16*D2‐BD) abolished transcriptional activity, while an N‐terminal mutant (*VaERF16*D3‐BD) still exhibited strong activity. We concluded that VaERF16 activates transcription through its C‐terminus, so *VaERF16*D1‐BD was selected for further experiments.

In our previous study, the transcript levels of *VvERF16* in grape leaves were found to be significantly up‐regulated upon *B*. *cinerea* challenge (Zhu et al., 2019). To investigate the potential role of *VaERF16* in regulating defence responses, we analysed its transcript levels in fruits of the *B. cinerea*‐resistant line Shuang You after inoculation with *B. cinerea*, in parallel with a similar analysis of *VvERF16* in *V. vinifera* ‘Red Globe’ (a *B. cinerea*‐susceptible line). The transcript levels of *VaERF16* were up‐regulated during the whole infection period compared to the mock‐inoculated control and reached a maximum at 3 days postinoculation (dpi). In the early infection stage (1 dpi), transcript levels of *VaERF16* were strongly up‐regulated in Shuang You, while they did not change in Red Globe (Figure S1a). The phytohormones methyl JA (MeJA) and ET have been shown to participate in defence against necrotrophs such as *B*. *cinerea* (Pieterse et al., 2009). To assess their potential relationship with *VaERF16*, we treated Shuang You leaves with each of the hormones. After treatment with ethephon, an ethylene‐releasing compound, transcript levels of *VaERF16* increased and peaked after 6 h, when they were 6‐fold higher than control levels. After MeJA treatment, transcript levels of *VaERF16* decreased after 6 and 12 h and then increased at the 24 and 48 h time points (Figure S1b,d). We also measured *ERF16* transcript abundance in different organs and found that the transcript levels of *VvERF16* were much higher in roots than in other organs, while the transcript levels of *VaERF16* were particularly high in leaves (Figure S1c).

### 2.2 Heterologous expression of *VaERF16* in *A. thaliana* enhances resistance to *B. cinerea*

**FIGURE 2:**
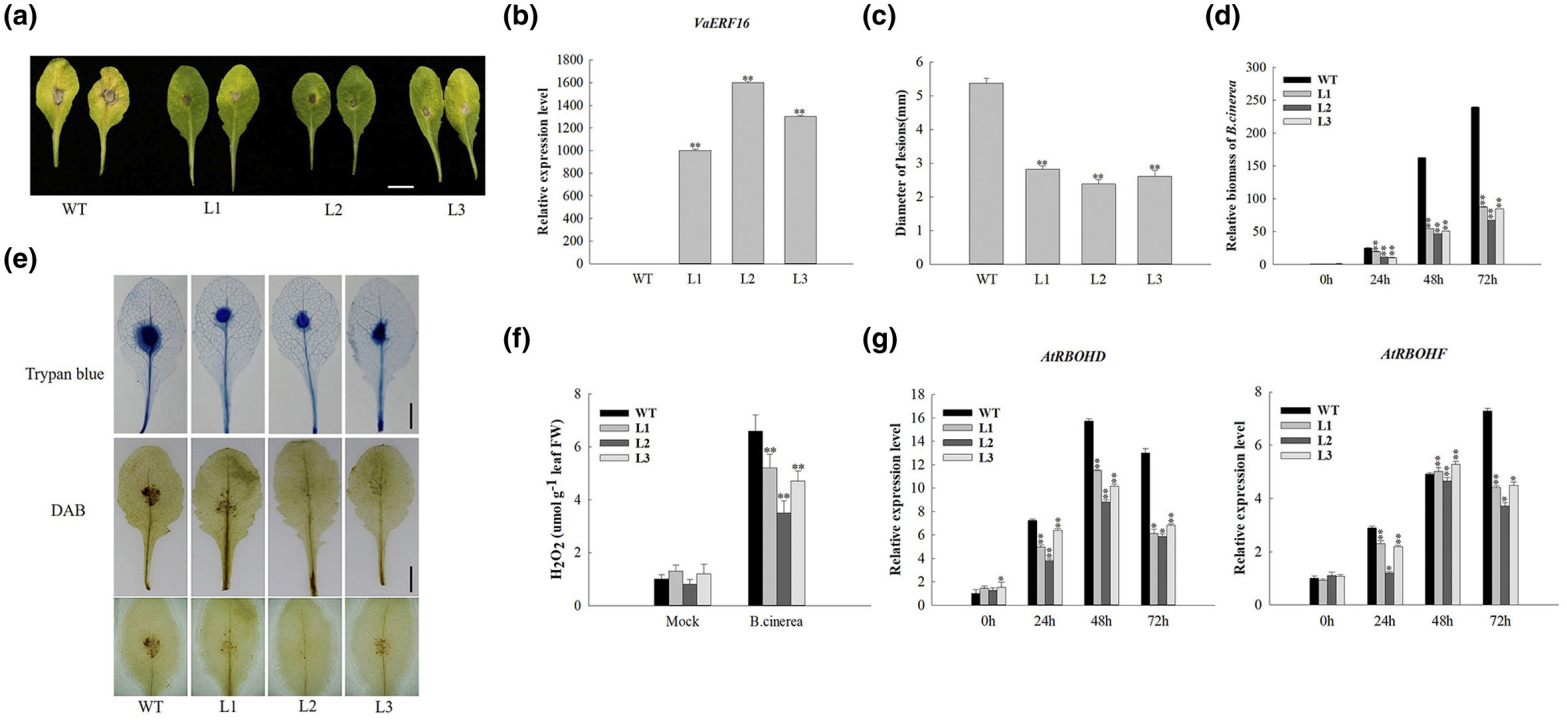
Overexpression of *VaERF16* in *Arabidopsis thaliana* enhances resistance to *Botrytis cinerea*. (a) Disease symptoms on *VaERF16* overexpressing (OE) lines (L1, L2, and L3) and wild‐type (WT) leaves 3 days postinoculation (dpi). Scale bar = 1 cm. (b) Relative gene expression of *VaERF16* in *A. thaliana* transgenic lines. (c) Diameter of *B. cinerea* lesions 3 dpi. (d) Quantitative PCR quantification of *B. cinerea* colonization. Total genomic DNA from *B. cinerea*‐infected leaves was isolated at 0, 24, 48, and 72 h after inoculation. *B*. *cinerea Actin* was used to determine *B. cinerea* biomass in infected plant tissues. (e) Trypan blue straining to visualize cell death. 3,3′‐Diaminobenzidine (DAB) staining for H_2_O_2_ detection. Leaves were collected 72 h after *B. cinerea* infection. Scale bar = 1 cm. (f) Measurement of the H_2_O_2_ content in leaves 72 h after infection with *B*. *cinerea*. (g) Gene expression analysis of the NADPH oxidase genes *AtRBOHD* and *AtRBOHF* in *Arabidopsis* transgenic lines and WT at 0, 24, 48, and 72 h after *B. cinerea* inoculation. *AtActin2* (AT3G18780), *EF1α* (AT5G60390), and *UBQ5* (AT3G62250) were used as internal reference genes. Error bars indicate the *SD* from three independent experiments. Statistical significance was determined by Student's two‐tailed *t* test (*p < 0.05, **p < 0.01)

**FIGURE 3:**
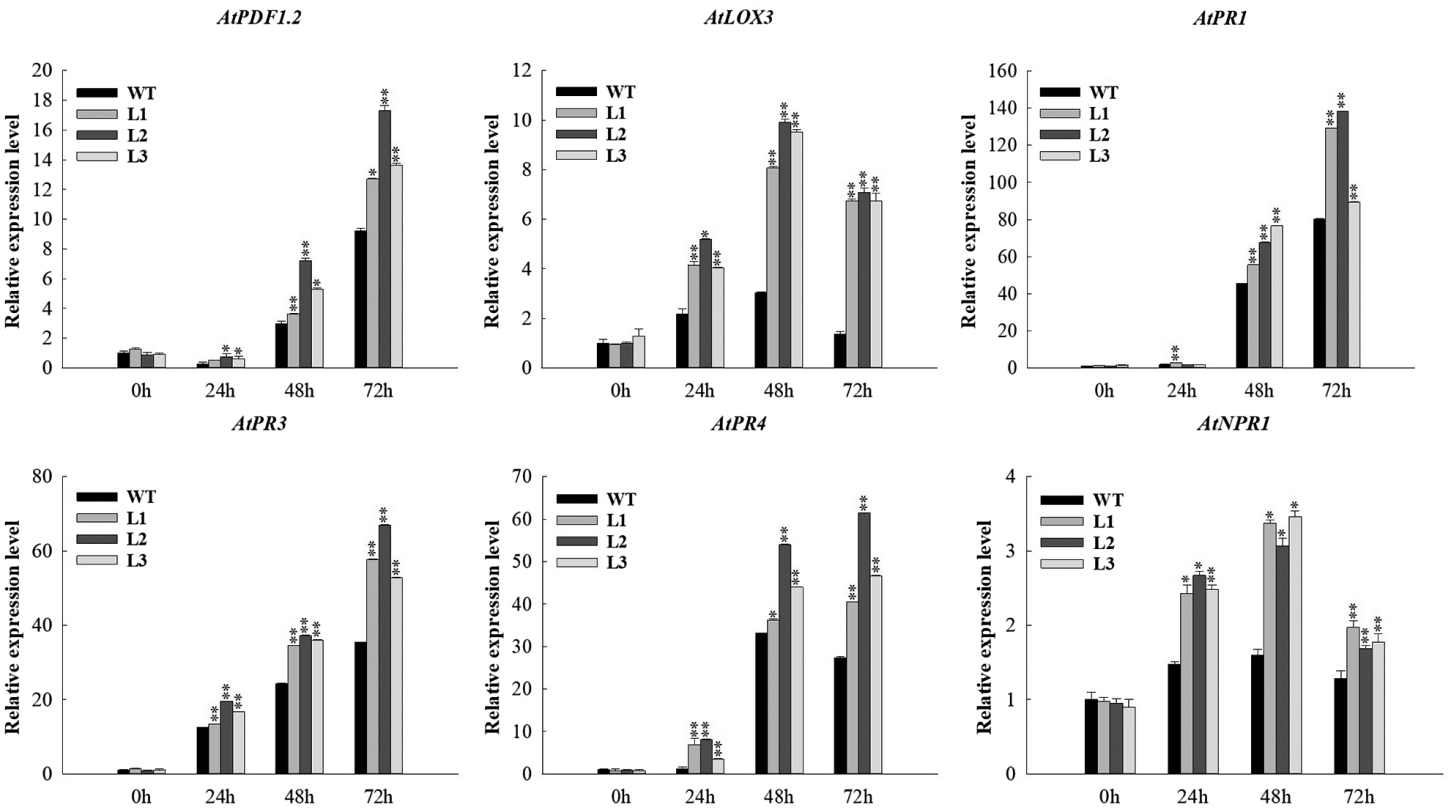
Reverse transcription‐quantitative PCR analysis of defence‐related genes in *VaERF16* overexpressing (OE) lines (L1, L2, and L3) and wild‐type (WT) plants 0, 24, 48, and 72 h after *Botrytis cinerea* inoculation. *AtActin2* (AT3G18780), *EF1α* (AT5G60390), and *UBQ5* (AT3G62250) were used as internal reference genes. Error bars indicate the *SD* from three independent experiments. Statistical significance was determined by Student's two‐tailed *t* test (*p < 0.05, **p < 0.01)

To further characterize the role of *VaERF16* in disease resistance, we generated *VaERF16*‐overexpressing *A. thaliana* lines. Three transgenic T_3_ generation lines (L1, L2, and L3) expressing *VaERF16* (Figure 2b), as well as Col‐0 (wild type [WT]) were inoculated with *B. cinerea*. WT plants leaves turned yellow and showed larger lesion diameters than the transgenic lines at 3 dpi (Figure 2a,c), and a quantification of *B. cinerea* colonization in infected leaves revealed less colonization in the transgenic lines (Figure 2d). Because ROS production is one of the earliest defence responses in the host plant interaction with *B. cinerea* (Asselbergh et al., 2007), we measured H_2_O_2_ accumulation using 3,3'‐diaminobenzidine (DAB) staining. The transgenic lines showed less ROS accumulation at 72 h postinoculation (hpi) than did WT plants (Figure 2e). We also quantified endogenous H_2_O_2_ content. The results showed that the H_2_O_2_ content in vivo was higher in WT lines at 72 hpi (Figure 2f). In addition, the transcript levels of the NADPH oxidase genes *AtRBOHD* and *AtRBOHF*, which are involved in ROS production (Chaouch et al., 2012; Kadota et al., 2015), were down‐regulated in transgenic plants and lower than those in WT plants especially at 72 hpi (Figure 2g). Moreover, a trypan blue assay indicated that the transgenic lines had less cell death than the WT plants at 72 hpi (Figure 2e). Microscopic observation revealed that at 24 hpi, *B. cinerea* conidia were already found on WT leaves, while almost no fungal growth was observed in the transgenic lines. From 24 to 72 hpi, there were numerous spreading lesions with mycelia and longer germ tubes on the WT leaves, while fewer conidia and shorter germ tubes were observed on the leaves of the transgenic lines (Figure S2). To investigate the relationship with phytohormone signalling, we analysed the transcript levels of two SA‐responsive genes (*AtPR1* and *AtNPR1*) and four JA/ET‐responsive genes (*AtPDF1.2*, *AtLOX3*, *AtPR3*, and *AtPR4*). We observed that the transcript levels of *AtPDF1.2*, *AtPR3*, and *AtPR4* were higher at 72 hpi compared with WT plants, while the transcript levels of *AtLOX3* increased at 24 hpi and peaked at 48 hpi, but then decreased at 72 hpi. The transcript levels of *AtNPR1* were up‐regulated at the early stage of infection and decreased at 72 hpi. In contrast, transcript levels of *AtPR1* showed no obvious induction at the early stage, but significantly increased at 48 and 72 hpi (Figure 3).

### 2.3 Heterologous expression of *VaERF16* in *A. thaliana* enhances resistance to *P. syringae* pv. *tomato* DC3000

The three transgenic lines and WT plants were infected with *P. syringae* pv. *tomato* (Pst) DC3000 to test a possible role for *VaERF16* in bacterial resistance. At 72 hpi, WT plants showed severe chlorosis, while almost no symptoms were apparent in the transgenic plants (Figure S3a). When the abundance of bacteria in leaves was measured, the levels were significantly higher in WT plants than in the transgenic plants (Figure S3b,e). Trypan blue assays and DAB staining also showed more cell death and ROS accumulation in WT plants at 72 hpi than in the transgenic lines (Figure S3c). Callose can act as a physical barrier to repress pathogen attack and contribute to plant immunity at the early stage of infection (Wang et al., 2018), and this can be visualized using aniline blue staining. We observed an increase in callose deposition at 24 h after Pst DC3000 inoculation in the transgenic plants, but not in WT plants (Figure S3d). Hemibiotrophic pathogens, such as Pst DC3000, are sensitive to defence responses regulated by SA (Pieterse et al., 2009) and we observed that the transcript levels of *AtPR1* and *AtNPR1* increased at 24 hpi and were significantly induced at 72 hpi in the transgenic plants. Moreover, two JA/ET‐responsive genes, *AtPR3* and *AtPR4*, showed similar expression patterns with a lower degree of up‐regulation compared to the WT control. In contrast, transcript levels of the JA/ET signalling‐related gene *AtPDF1.2* decreased at 24 hpi and increased at 48 until 72 hpi, and the transcript levels of *AtLOX3* increased at 24 hpi and decreased at 72 hpi in the transgenic plants compared to WT plants (Figure S3f).

### 2.4 VaERF16 interacts with VaMYB306

**FIGURE 4:**
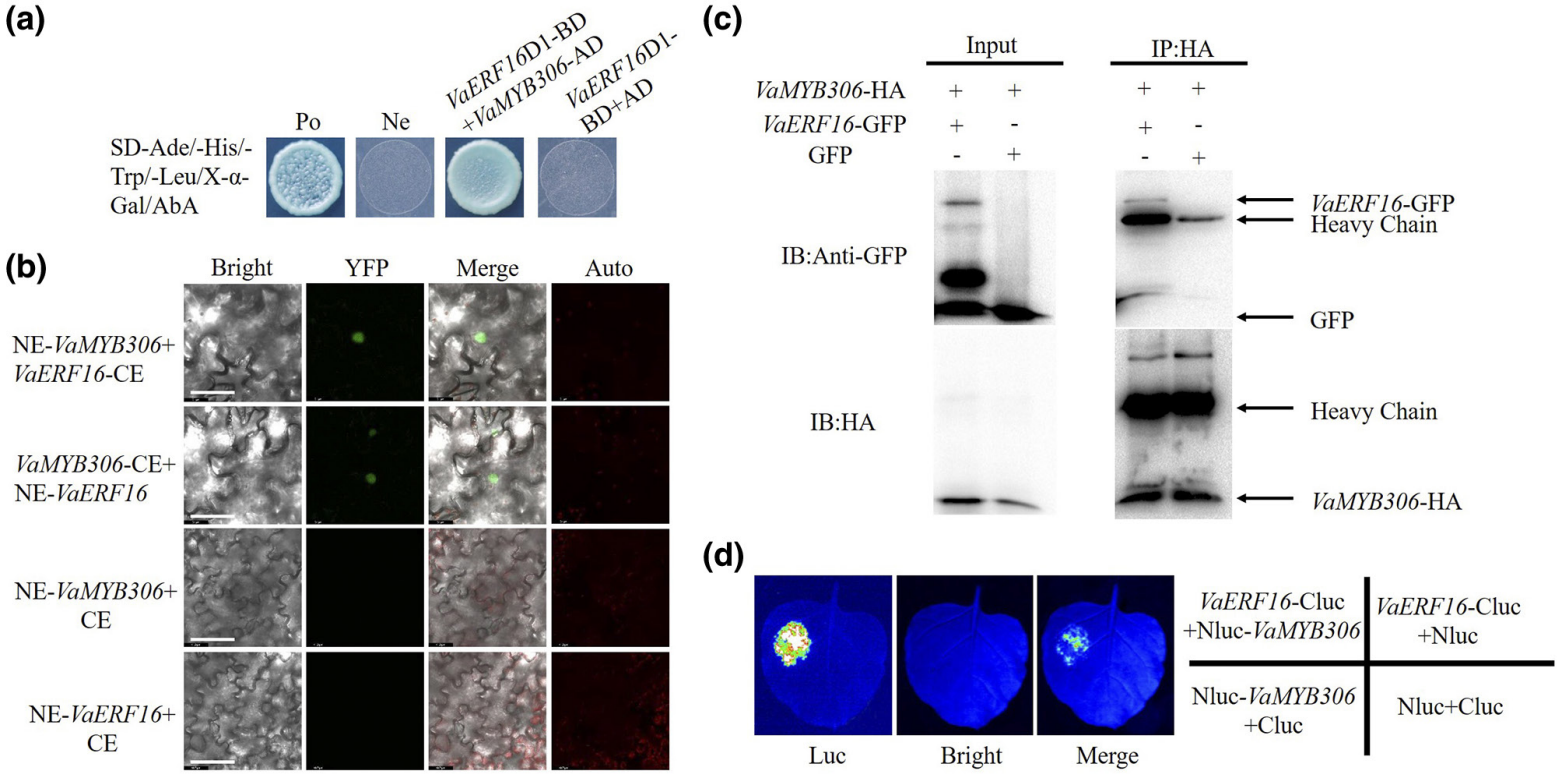
VaERF16 interacts with VaMYB306. (a) Yeast two‐hybrid assay. pGBKT7‐*VaERF16*D1 and pGADT7‐*VaMYB306* plasmids were co‐transformed into Y2H Gold cells. pGBKT7‐p53 + pGADT7‐T and pGBKT7‐Lam + pGADT7‐T served as positive and negative controls, respectively. (b) In vivo bimolecular fluorescence complementation assay. Merged fluorescent and visible light images. Scale bar = 60 μm. Auto indicates chloroplast autofluorescence. (c) Co‐immunoprecipitation assay. VaMYB306‐HA/GFP and VaERF16‐GFP/VaMYB306‐HA were co‐expressed in tobacco leaves. Anti‐HA antibodies were used to immunoprecipitate VaERF16‐GFP. Gel blots were probed with anti‐GFP or anti‐HA antibodies. (d) The VaERF16 and VaMYB306 interaction was confirmed using a split luciferase assay with *Nicotiana benthamiana* leaves. pCB1300‐*VaERF16*‐Cluc/pCB1300‐Nluc, pCB1300‐Nluc*‐VaMYB306*/pCB1300‐Cluc, and pCB1300‐Cluc/pCB1300‐Nluc were used as controls

To further elucidate the resistance mechanism of *VaERF16*, a Y2H assay was used to identify candidate interacting proteins. *VaERF16*D1‐BD was used as bait to screen a cDNA library derived from Shuang You leaves challenged with *B*. *cinerea*. A total of seven clones were obtained (Table 1), three of which contained the same sequence, encoding VaMYB306 (GenBank accession no. XP_002283575). *VaMYB306* belongs to the R2R3‐MYB gene family, and its *A*. *thaliana* homologue (*AtMYB30*) has been shown to be a positive regulator of the hypersensitive cell death programme in response to pathogen attack (Vailleau et al., 2002). Accordingly, we selected *VaMYB306* as a target for further analysis. Yeast colonies harbouring both pGBKT7‐*VaERF16*D1 and pGADT7‐*VaMYB306* grew on SD/−Ade−His−Leu−Trp medium and showed blue colouration in the presence of X‐α‐Gal and AbA, similar to the positive control (Figure 4a). These results suggest that VaERF16 interacts specifically with VaMYB306 in yeast cells.

**TABLE 1:** Positive clones obtained from a cDNA library of Chinese wild grape *Vitis amurensis* ‘Shuang You’ after *Botrytis cinerea* infection using *VaERF16* as bait

Accession number	Protein name	Number of clones	Description
XP_002264659	Digalactosyldiacylglycerol synthase 1	1	Digalactosyldiacylglycerol biosynthesis
XP_002277703	Eukaryotic initiation factor 4A‐8	1	Involved in ATP‐dependent RNA unwinding.
XP_002283575	MYB‐related protein 306	3	In response to pathogen attack
XP_002263448	Chaperone protein DnaJ GFA2	1	Prevents the aggregation of unfolded substrate and forms a ternary complex with both substrate and DnaK/Hsp70
XP_010647098	Polyphenol oxidase	1	Participates in scavenging of reactive oxygen species

To verify the interaction in planta, we performed bimolecular fluorescence complementation (BiFC) (Bracha‐Drori et al., 2004; Walter et al., 2004) in tobacco leaves. As shown in Figure 4b, in contrast to the control samples, the combinations of *VaERF16*‐CE/NE‐*VaMYB306* and NE‐*VaERF16*/*VaMYB306*‐CE gave a fluorescent signal in the nucleus. We next used anti‐HA and anti‐green fluorescent protein (GFP) antibodies for co‐immunoprecipitation (Co‐IP) to test the interaction between VaERF16 and VaMYB306 and observed that VaMYB306‐HA co‐precipitated with VaERF16‐GFP but not with the control GFP (Figure 4c). A split luciferase assay was performed in *N. benthamiana* leaves, and luciferase activity was detected in leaves co‐transformed with Nluc‐*VaERF16* and *VaMYB306*‐Cluc, while control samples showed no luciferase activity (Figure 4d). Taking these results into account, we conclude that VaERF16 and VaMYB306 interact with each other and are co‐localized in the nucleus.

### 2.5 Bioinformatics analysis and *VaMYB306* expression profiles

To date, the R2R3‐MYB gene family has been widely studied in *A. thaliana* (Katiyar et al., 2012), soybean (Du et al., 2012), apple (Liu et al., 2019), alfalfa (Zhou et al., 2019), tomato (Li et al., 2016), cotton (Wang et al., 2019), and tobacco (Liu et al., 2016). Thus, we compared MYB proteins and their homologues in these seven different plant species by multiple sequence alignment. The results showed that VaMYB306 contains highly conserved canonical R2 and R3 MYB domains (Figure S4a). A subsequent phylogenetic analysis showed it is closely related to apple MdMYB306 (GenBank no. XP_028953285.1) and cotton GhMYB306 (GenBank no. XP_040970228.1) (Figure S4b). Consistent with its presumed function as a TF, a VaMYB306‐GFP fusion protein was localized in the nucleus (Figure S4c). We also analysed *MYB306* gene expression patterns in different organs of Red Globe and Shuang You and found that it reached high transcript levels in leaves and fruits in both cultivars (Figure S4d). When Red Globe fruits were inoculated with *B*. *cinerea*, the transcript levels of *VvMYB306* increased up to 3‐fold at 1 dpi compared with the control, after which it decreased at 3 dpi and 5 dpi. Similarly, transcript levels of *VaMYB306* increased 2.5‐fold at 1 dpi in Shuang You (Figure S5a). However, the transcript levels of *VvMYB306* increased at 4 h and were up to 3‐fold higher at 8 h in leaves of Red Globe after *B*. *cinerea* inoculation, while the transcript levels of *VaMYB306* showed no significant difference at 4 and 8 h, but were up‐regulated at 18 h and then peaked at 36 h, increasing 8‐fold in Shuang You (Figure S5c). To investigate whether *VaMYB306* participates in phytohormone signalling pathways, we treated Shuang You leaves with MeJA and ethephon. Compared with the control, transcript levels of *VaMYB306* showed no change soon after ethephon treatment, with transcript levels significantly increased by 5‐fold after 12 h, before decreasing at 24 and 48 h (Figure S5b). After MeJA treatment, *VaMYB306* transcript levels decreased at 0.5 h but were up‐regulated at 1, 3, and 6 h and peaked at 12 h, being 4.5‐fold higher than control levels (Figure S5d).

### 2.6 VaERF16 binds to the *VaPDF1.2* promoter and increases its transcript levels by interacting with VaMYB306

**FIGURE 5:**
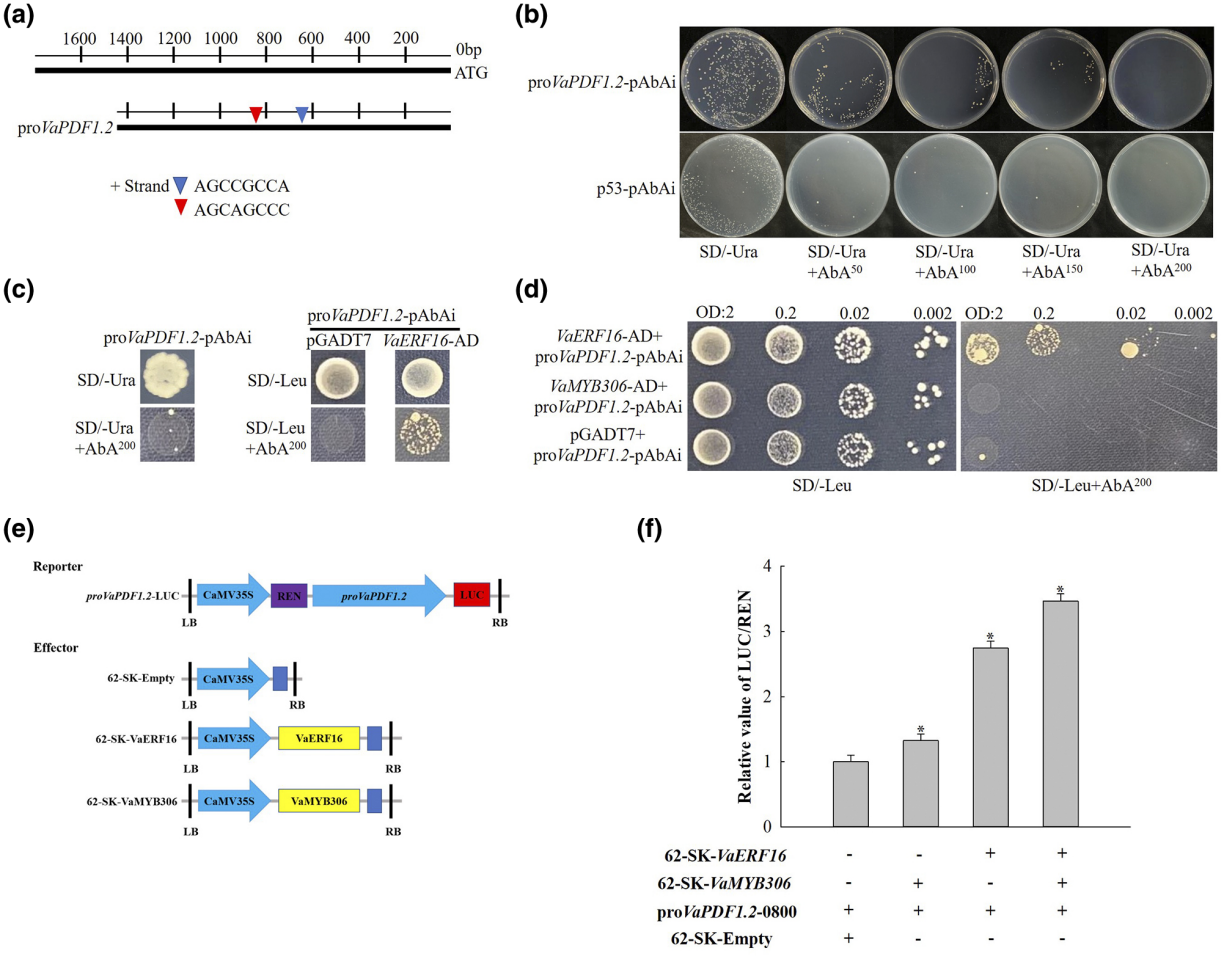
VaERF16 binds to the *VaPDF1.2* promoter and enhances its transcriptional levels by interacting with VaMYB306. (a) Graphical representation of the *VaPDF1.2* promoter. The GCC box (AGCCGCCA) and DNA‐binding site (AGCAGCCC) are ERF‐binding *cis*‐elements. (b) Determination of the minimum inhibitory concentration of aureobasidin A (AbA) for the bait (pro*VaPDF1.2*‐pAbAi). p53‐pAbAi was used as a control. (c) Yeast one‐hybrid assay indicating that VaERF16 can directly bind to the *VaPDF1.2* promoter. The pGADT7 vector was used as a control. Yeast strains containing pro*VaPDF1.2*‐pAbAi were used as baits. Yeast cultures were inoculated on SD/−Leu medium containing 200 ng/ml AbA. (d) Yeast one‐hybrid experiment indicating that VaERF16 can interact with the *VaPDF1.2* promoter, while VaMYB306 cannot. Strains harbouring the *VaPDF1.2* promoter were used as baits. The pGADT7 vector was used as a control. (e) Schematic diagram of the effector and reporter constructs used for the dual‐luciferase assay. (f) Luciferase (LUC) assay indicating that VaERF16 increases *VaPDF1.2* promoter activity. VaERF16 coexpressed with VaMYB306 showed stronger activation of the *VaPDF1.2* promoter. The LUC/REN ratio of the empty vector 62‐SK and the *VaPDF1.2* promoter was used for normalization. Data are presented as the mean ± *SD* of three independent experiments, with three replicates in each experiment. Asterisks represent significant differences (*p < 0.05)

The interaction between JA and ET signalling during the defence response is synergistic, and ERF proteins bind specifically to DNA sequences containing GCC boxes, which are generally present in the promoters of JA‐ and ET‐inducible defence genes (Hao et al., 1998, 2002). For example, in *A. thaliana*, the promoter region of the defensin gene *PDF1.2*, which is a key defence gene functioning downstream of the JA and ET signalling pathways (Penninckx et al., 1998), contains two GCC box elements that are direct targets of ERF proteins such as ERF1, ORA59, and ERF96 (Huang et al., 2016). Because *VaERF16* plays important roles in the JA and ET signalling pathways (Figures S1b,d and 3), we speculated that it may also act as a regulator of *PDF1.2*. To test this, the *VaPDF1.2* promoter (GenBank accession no. XM_002272877) was cloned from the Shuang You genomic DNA sequence. The *VaPDF1.2* promoter sequence contains a GCC box (AGCCGCCA) and another predicted binding site (AGCAGCCC) that may be recognized by VaERF16 (Figure 5a). We then conducted a yeast one‐hybrid assay to determine whether VaERF16 can bind to the *VaPDF1.2* promoter, using empty vector as a negative control. After determining the minimum inhibitory AbA concentration (200 ng/ml) (Figure 5b), VaERF16 was observed to directly bind to the *VaPDF1.2* promoter even at low concentrations, while VaMYB306 did not (Figure 5c,d). Dual‐luciferase assays were used then to determine whether the VaERF16–VaPDF1.2 interaction results in gene activation or suppression. The *VaPDF1.2* promoter was cloned into the pGreenII 0800‐LUC vector and co‐transformed into tobacco leaves with *VaERF16*, *VaMYB306*, or a combination of *VaERF16*/*VaMYB306* (Figure 5e). We observed that VaERF16 induced the accumulation of *VaPDF1.2* transcripts, with an increase in transactivation of almost 2.5‐fold, while VaMYB306 showed a limited effect. Notably, the combination of VaERF16 and VaMYB306 up‐regulated the transcript levels of *VaPDF1.2* in vivo (Figure 5f). These results suggest that VaERF16 can directly bind to the promoter of *VaPDF1.2* and that a VaERF16–VaMYB306 transcriptional complex contributes to pathogen defence by increasing the transcript levels of *VaPDF1.2*.

### 2.7 Transient *VaERF16* and *VaMYB306* overexpression enhances *B. cinerea* resistance in two disease‐susceptible grape varieties

**FIGURE 6:**
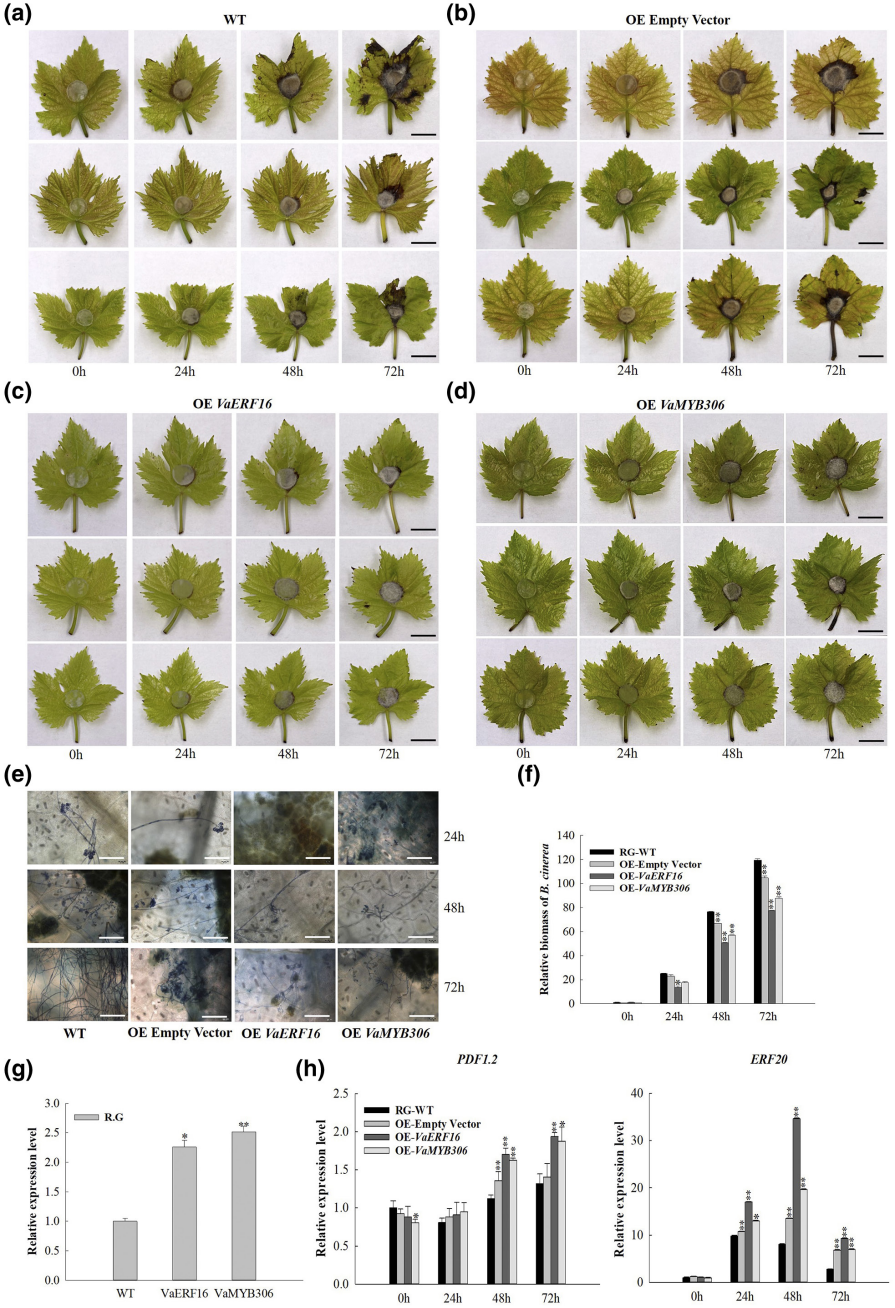
Transient overexpression of *VaERF16* or *VaMYB306* in Red Globe leaves enhances resistance to *Botrytis cinerea*. (a–d) Disease symptoms on infiltrated leaves (wild‐type [WT,] empty overexpression [OE] vector, *VaERF16* OE, and *VaMYB306* OE) after *B. cinerea* inoculation. Scale bar = 1 cm. Each row of photographs represents an independent experiment. (e) Trypan blue staining to detect the development of *B*. *cinerea* conidia. Scale bar = 150 μm. The leaves were collected at 24, 48, and 72 h after inoculation. (f) Quantitative PCR (qPCR) quantification of *B. cinerea* colonization. Total genomic DNA from *B. cinerea*‐infected leaves was isolated at 0, 24, 48, and 72 h after inoculation. *B*. *cinerea Actin* was used to determine *B. cinerea* biomass in infected plant tissues. (g) Reverse transcription‐qPCR analysis of *VaERF16* and *VaMYB306* in infiltrated leaves. Asterisks represent significant differences between infiltrated leaves (*VaERF16* OE, *VaMYB306* OE) and WT leaves. (h) Expression profiles of the defence‐related genes *PDF1.2* and *ERF20* in infiltrated leaves after inoculation. *ACTIN7* (XM_002282480), *GAPDH* (XM_002278316.4), and *EF1‐α* (XM_002284888) were used as internal reference genes. Error bars indicate the *SD* from three independent experiments. Asterisks represent significant differences (*p < 0.05, **p < 0.01, Student's two‐tailed *t* test)

To better understand how *VaERF16* and *VaMYB306* function in the pathogen response, we separately transiently transformed the two genes into susceptible grape (Red Globe and Thompson Seedless) leaves and inoculated them with an agar disc containing *B. cinerea* mycelia. The transcript levels of *VaERF16* and *VaMYB306* were analysed at 48 h after infiltration. They were 2–3‐fold higher than in untransformed leaves, indicating that *VaERF16* and *VaMYB306* were successfully overexpressed (Figures 6g and S6g). The lesions caused by *B*. *cinerea* on WT leaves, and empty overexpression (OE) vector control leaves were significantly larger than leaves from *VaERF16* OE and *VaMYB306* OE plants. Moreover, WT leaves and empty vector control leaves had fully decayed and were covered with *B. cinerea* mycelia at 72 hpi (Figures 6a–d and S6a–d). Hyphae of *B. cinerea* were observed on WT and empty vector control leaves at 24 hpi, while only few conidia were observed on *VaERF16* OE and *VaMYB306* OE leaves. In addition, at 72 hpi, fewer hyphae were found on *VaERF16* OE and *VaMYB306* OE leaves compared to controls in both cultivars (Figures 6e and S6e). Moreover, a quantification of *B. cinerea* biomass in infected leaves revealed less colonization in *VaERF16* OE and *VaMYB306* OE leaves (Figures 6f and S6f). The transcript levels of *PDF1.2* increased at 24 hpi and peaked at 72 hpi, whereas no such change was observed in WT plants and empty vector control plants after *B*. *cinerea* infection in Thompson Seedless (Figure S6h). However, in Red Globe, the transcript levels of *PDF1.2* showed no difference in *VaERF16* OE and *VaMYB306* OE leaves at 24 hpi compared to WT and empty vector control plants, but were higher at 48 and 72 hpi (Figure 6h).

*ERF1* has been reported to function as a regulator of resistance to *B*. *cinerea* and to integrate signals from the JA and ET signalling pathways in *A. thaliana* (Gutterson & Reuber, 2004; Huang et al., 2016). Thus, the expression profile of the grape homologue *ERF20* was also analysed in both grape cultivars. Transcript levels of *ERF20* increased 2‐fold at 24 hpi in *VaERF16* OE and *VaMYB306* OE leaves compared with the control, but were significantly lower at the late infection stages in Thompson Seedless (Figure S6h). In Red Globe, transcript levels of *ERF20* were increased in the *VaERF16* OE and *VaMYB306* OE leaves compared with the control at 24 hpi, peaked at 48 hpi after *B*. *cinerea* inoculation, and were significantly down‐regulated at 72 hpi (Figure 6h). This suggests that overexpression of *VaERF16* and *VaMYB306* enhanced resistance to *B*. *cinerea* via the JA/ET signalling pathway in susceptible grape varieties.

### 2.8 Transient silencing of *VaERF16* and *VaMYB306* reduces *B. cinerea* resistance in two disease‐resistant grape varieties

**FIGURE 7:**
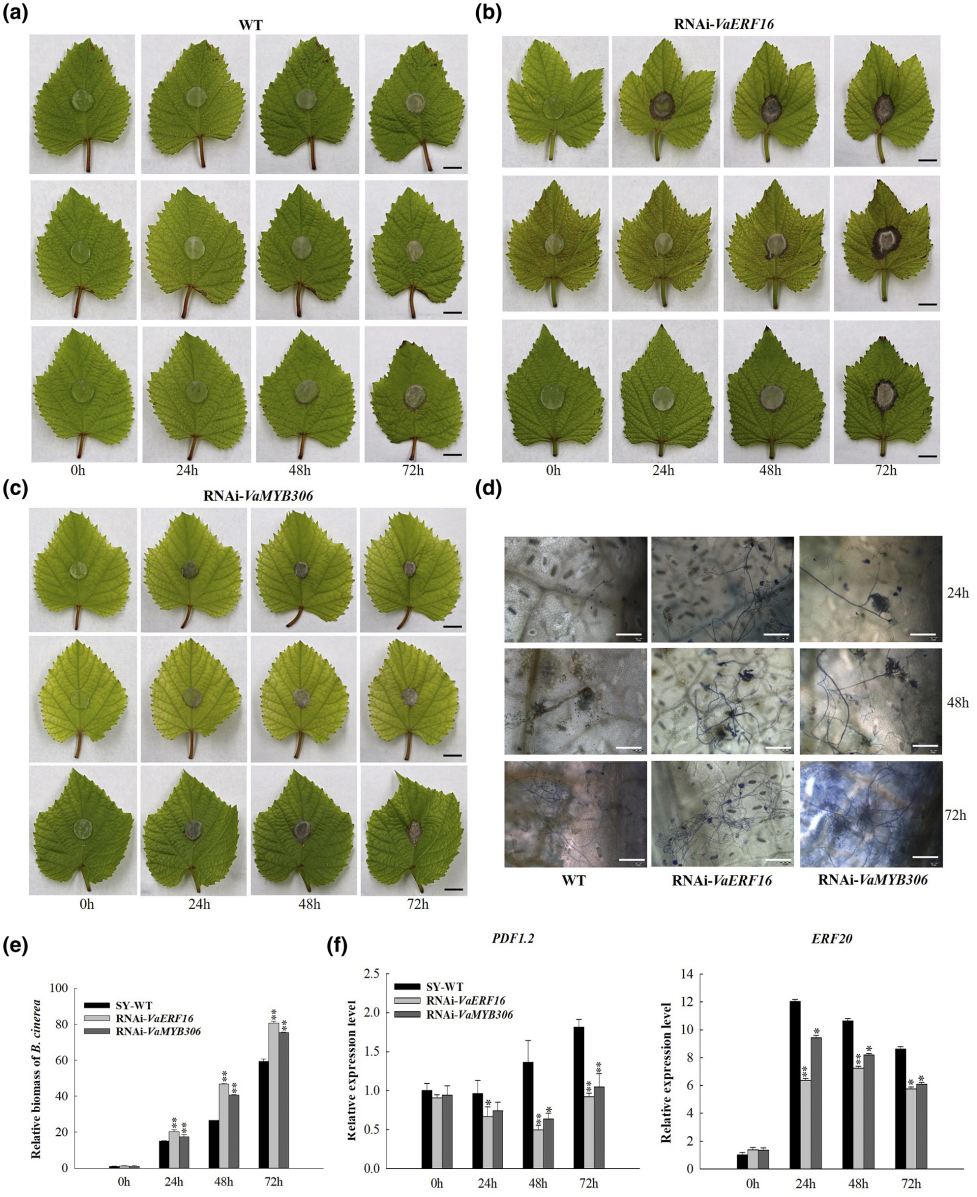
Transient silencing of *VaERF16* and *VaMYB306* in leaves of *Vitis amurensis* 'Shuang You' reduces resistance to *Botrytis cinerea*. (a–c) Disease symptoms on infiltrated leaves (wild‐type [WT], *VaERF16*‐RNAi, and *VaMYB306*‐RNAi) after *B*. *cinerea* inoculation. The leaves were sampled at 0, 24, 48, and 72 h postinoculation. Scale bar = 1 cm. Each row of photographs represents an independent experiment. (d) Trypan blue staining to detect the development of *B*. *cinerea* conidia. Scale bar = 150 μm. (e) Quantitative PCR quantification of *B. cinerea* colonization. Total genomic DNA from *B. cinerea*‐infected leaves was isolated at 0, 24, 48, and 72 h after inoculation. *B*. *cinerea Actin* was used to determine *B. cinerea* biomass in infected plant tissues. (f) Expression profiles of the defence‐related genes *PDF1.2* and *ERF20* in infiltrated leaves after inoculation. *ACTIN7* (XM_002282480), *GAPDH* (XM_002278316.4), and *EF1‐α* (XM_002284888) were used as internal reference genes. Error bars indicate the *SD* from three independent experiments. Asterisks represent significant differences (*p < 0.05, **p < 0.01, Student's two‐tailed *t* test)

Previous studies revealed that *V. quinquangularis* 'Ju Meigui' is highly resistant to *B*. *cinerea* (Rahman et al., 2020). Thus, we next used an RNA interference (RNAi) approach to repress transcript levels of *VaERF16* and *VaMYB306* in transiently transformed Ju Meigui and Shuang You leaves. The transcript levels of *VaERF16* were reduced to 30% and those of *VaMYB306* to 70% of the levels in WT Ju Meigui leaves (Figure S8b). In Shuang You leaves, the transcript levels of *VaERF16* were reduced to 30% and those of *VaMYB306* to 50% of nontransgenic levels (Figure S8a). After inoculation with *B*. *cinerea*, the *VaMYB306*‐RNAi leaves that touched the agar disc containing *B. cinerea* mycelia were necrotic by 24 hpi, and the lesions on *VaERF16*‐RNAi leaves were much larger than those on leaves of WT plants, especially at 72 hpi, in both grape cultivars (Figures 7a–c and S7a–c). Moreover, compared to WT Ju Meigui leaves, more mycelia were observed on *VaERF16*‐RNAi and *VaMYB306*‐RNAi leaves, especially at 72 hpi (Figure S7d). Large numbers of mycelia were found on *VaERF16*‐RNAi and *VaMYB306*‐RNAi leaves at 24 and 48 hpi, while fewer *B. cinerea* conidia were found on WT leaves of Shuang You at the same stage (Figure 7d). In addition, *B. cinerea* biomass was lower on WT leaves than on *VaERF16*‐RNAi and *VaMYB306*‐RNAi leaves at 72 hpi, indicating that *VaERF16*‐RNAi and *VaMYB306*‐RNAi plants exhibited enhanced disease susceptibility to *B. cinerea* (Figures 7e and S7e). Furthermore, transcript levels of *PDF1.2* and *ERF20* were similar in both cultivars, but were lower in *VaERF16*‐RNAi and *VaMYB306*‐RNAi leaves compared to WT leaves at different time points of infection (Figures 7f and S7f). Taken together, these results are consistent with the observed increased resistance of *VaERF16* OE or *VaMYB306* OE leaves and the sensitivity of *VaERF16*‐RNAi or *VaMYB306*‐RNAi leaves to *B*. *cinerea* attack, indicating that *VaERF16* and *VaMYB306* contribute to disease resistance against this pathogen.

## 3 DISCUSSION

In previous studies, members of the ERF TF family have been widely reported to play roles in disease resistance (Berrocal‐Lobo et al., 2002; Gutterson & Reuber, 2004; Moffat et al., 2012). In grape, 113 ERF genes have been identified and divided into 12 groups, and expression profiling has shown that the expression of *ERF16*, which belongs to group VII, is strongly induced after inoculation with *B*. *cinerea* (Zhu et al., 2019), suggesting that *ERF16* is important for grape tolerance to *B*. *cinerea*. Thus, we determined the potential functions of *VaERF16* in response to pathogen attack and the underlying mechanisms.

Recent studies have shown that many ERF genes play roles in the regulation of *B. cinerea* resistance in various plant species. In *N*. *benthamiana*, overexpression of *NbERF173* enhanced *B*. *cinerea* resistance while silencing of *NbERF173* enhanced susceptibility (Yu et al., 2020). In tomato, silencing of *SlERF.A1*, *SlERF.A3*, *SlERF.B4*, or *SlERF.C3* resulted in increased susceptibility to *B. cinerea* (Ouyang et al., 2016). In *Arabidopsis*, constitutive overexpression of *AtERF5*, *AtERF6*, *AtERF15*, and *AtERF152* also resulted in *B*. *cinerea* resistance (Moffat et al., 2012; Pillai et al., 2020; Zhang et al., 2015), and *AtERF72* was also shown to positively regulate *B. cinerea* resistance (Li et al., 2021). Similarly, in the present study, the transcript levels of *VaERF16*, which is an *AtERF72* homologue, were significantly increased in fruits of Shuang You during the whole *B. cinerea* infection period (Figure S1a), in agreement with our previous study that showed that *ERF16* expression was induced in grape leaves inoculated with *B. cinerea* (Zhu et al., 2019). Moreover, overexpression of *VaERF16* in *Arabidopsis* and grape enhanced the resistance to *B. cinerea* compared with WT plants (Figures 2, 6, 7, S6, and S7). These results suggested that *VaERF16* positively modulates immunity against *B. cinerea*.

The SA signalling pathway is associated with biotrophic pathogen attack, while the JA/ET signalling pathway is connected to attacks from necrotrophic pathogens (Pieterse et al., 2012); ERF genes contribute to immune responses through both these pathways (Zang et al., 2021). In our study, the transcript levels of *VaERF16* increased after treatments with MeJA and ethephon at different time points (Figure S1b,d), suggesting that *VaERF16* may be involved in JA/ET‐related defence signalling. Moreover, overexpression of *VaERF16* in *Arabidopsis* enhanced the resistance to *B*. *cinerea* compared to WT plants (Figure 2) and the transcript levels of four JA/ET signalling‐related defence genes were up‐regulated, while the SA signalling‐related gene *AtNPR1* showed no significant induction in response to *B*. *cinerea* (Figure 3). The transcript levels of key genes in the JA/ET signalling pathway also increased after infection in grape leaves transiently overexpressing *VaERF16*, while silencing resulted in their down‐regulation (Figures 6h, 7f, S6h, and S7f). When transgenic *A. thaliana* lines were inoculated with Pst DC3000, the transcript levels of SA signalling‐related genes showed a clear increase, especially at 72 hpi (Figure S3f), while transcript levels of JA/ET signalling‐related genes showed no change. These results indicate that overexpression of *VaERF16* enhanced resistance to Pst DC3000 via the SA signalling pathway.

ERF proteins can function in plant immunity through interactions with other proteins (Dong et al., 2015; Meng et al., 2013). For example, MdERF100 from apple interacts with MdbHLH92 to improve the resistance to powdery mildew (Zhang et al., 2021), and AtERF72 from *A. thaliana*, which is related to RAP2.3, was found to interact with ACBP4 to mediate defences (Li et al., 2008) and to directly interact with TGA4 to enhance disease resistance (Büttner & Singh, 1997). Finally, ORA59 was shown to enhance resistance against *Pectobacterium carotovorum* by interacting with AtERF72 (Kim et al., 2018). Interestingly, *AtERF72* is a gene highly homologous to *VaERF16*. These results suggest that *VaERF16* may also regulate plant immune responses to pathogens through interacting with other proteins. Here, we determined by Y2H, BiFC, Co‐IP, and split luciferase assays that VaERF16 interacts with VaMYB306 (Figure 4). A previous study revealed that *AtMYB30*, which is a *VaMYB306* homologue, acts as a positive regulator of the hypersensitive cell death programme in response to pathogen attack (Vailleau et al., 2002). We found that *B*. *cinerea* inoculation of grape increased transcript levels of *VaMYB306* 6‐fold at 72 hpi in leaves of Shuang You compared with the control (Figure S5c). Moreover, the transcript levels of *VaMYB306* were increased by MeJA and ET (Figure S5b,d). After infection by *B*. *cinerea*, leaves overexpressing *VaMYB306* showed enhanced resistance, while *VaMYB306* silencing increased susceptibility. Consistent with the leaf phenotypes, transcript levels of defence‐related genes were up‐ or down‐regulated (Figures 6, 7, S6, and S7). This suggested that *VaMYB306* increases resistance to *B. cinerea* and is regulated by the JA/ET signalling pathways.

Several ERF genes act as transcriptional activators to regulate plant immunity by binding to GCC box elements. For example, ERF68 enhances resistance to pathogens in tomato and tobacco leaves through directly binding to the GCC box of defence‐related genes (Liu & Cheng, 2017). Co‐IP analysis revealed that an ERF protein named DEWAX directly interacts with a GCC box element in the *PDF1.2a* promoter and increases *B. cinerea* tolerance in *A*. *thaliana* and *Camelina sativa* (Ju et al., 2017). Similarly, ERF96 from *A. thaliana* increases the transcript levels of the JA/ET defence‐related genes by binding to GCC motifs in their promoters, thereby enhancing resistance to necrotrophic pathogens (Catinot et al., 2015). Biochemical assays revealed that ERF11 binds to the GCC box of the *BT4* promoter during the *BT4*‐regulated *Arabidopsis* defence response to hemibiotrophic bacterial pathogens (Zheng et al., 2019), and in maize, ZmERF061 and ZmERF105 function as transcriptional activators by specifically binding to GCC box elements (Zang et al., 2020, 2021). In the present research, we identified a GCC box (AGCCGCCA) and a possible DNA‐binding sequence (AGCAGCCC) in the *VaPDF1.2* promoter and found that VaERF16, but not VaMYB306, bound to the *VaPDF1.2* promoter (Figure 5). Our luciferase assay revealed that *VaERF16* and *VaMYB306* alone increased *VaPDF1.2* promoter activity 1.2‐fold and 2.2‐fold, respectively, but their combined effect was a 3.5‐fold activation (Figure 5f). Taken together, our results suggest that VaMYB306 regulates the transcriptional levels of defence‐related genes as part of a complex with VaERF16.

**FIGURE 8:**
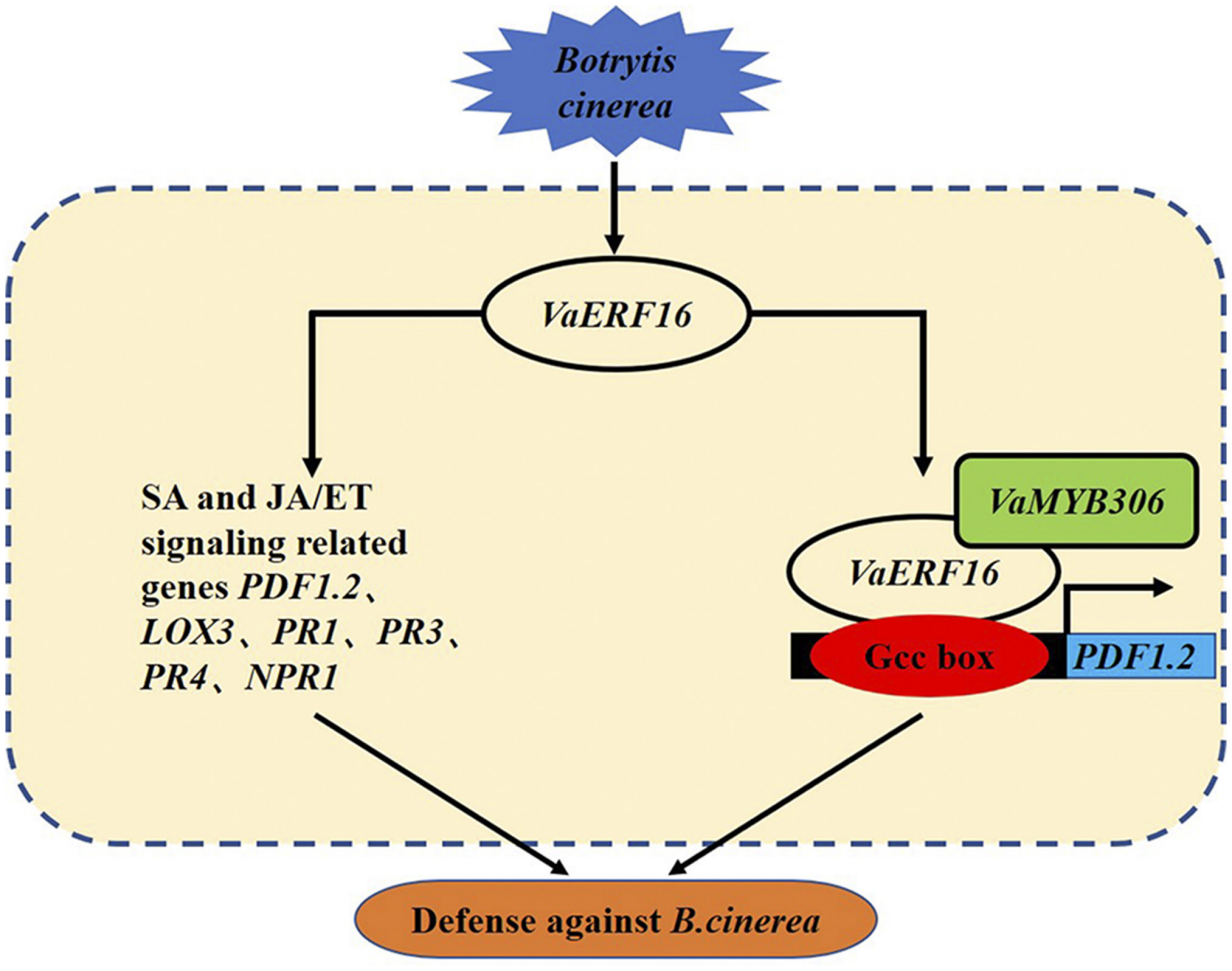
Model of disease resistance regulation by the ERF transcription factor VaERF16. Overexpression of *VaERF16* in *Arabidopsis thaliana* and grape leaves enhances the resistance to *Botrytis cinerea* via the salicylic acid (SA) and jasmonic acid (JA)/ethylene (ET) signalling pathways and increases the transcript levels of defence‐related genes. VaERF16 interacts with VaMYB306 to increase the transcript levels of *VaPDF1.2* by directly binding to its promoter. Arrows represent positive effects

In conclusion, we propose a model wherein *VaERF16* enhances resistance to *B*. *cinerea* via the SA and JA/ET signalling pathways. VaMYB306 participates in disease resistance by interacting with VaERF16, which form a complex and bind to elements in the promoters of defence‐related genes, including the GCC box in the *VaPDF1.2* promoter (Figure 8). Our data provide new insights into the functions and mechanisms of ERF genes in response to pathogen inoculation and indicate opportunities for enhancing grapevine disease resistance through breeding or genome modification strategies.

## 4 EXPERIMENTAL PROCEDURES

### 4.1 Plant materials and growth conditions

*V. quinquangularis* ‘Ju Meigui’, *V*. *amurensis* ‘Shuang You‘, and *V*. *vinifera* ‘Red Globe’ and ‘Thompson Seedless’ were obtained from the grapevine germplasm resources repository at Northwest A&F University, Yangling, Shaanxi, China. *N*. *benthamiana* was cultivated in a growth chamber at 25°C. *A. thaliana* ecotype Col‐0 and transgenic lines were cultivated in a plant incubator at 22°C under a 16‐h light/8‐h dark photoperiod at 70% relative humidity.

### 4.2 Pathogen inoculation and hormone treatments

*B. cinerea* was isolated from Red Globe and cultured on potato glucose agar for 3 weeks. Red Globe and Shuang You leaves and fruits were infected with *B*. *cinerea* as previously described (Wang et al., 2015, 2018). After inoculation, all leaves and fruits were stored at 22°C with a humidity of 90%–100% in the dark for 24 h, followed by a 12‐h light/12‐h dark photoperiod. Control samples were sprayed with distilled water. Leaves were collected at 4, 8, 18, and 36 hpi and fruits were collected at 0, 1, 3, and 5 dpi for further analysis.

MeJA treatment was carried out by spraying Shuang You leaves with 50 μM MeJA. For ET treatment, ethephon (C_2_H_6_CIO_3_P) was diluted with double distilled water to 0.5 g/L and then sprayed onto leaves. Leaves were collected 0, 0.5, 1, 3, 6, 12, 24, and 48 h after treatment (Li et al., 2010). Leaves sprayed with sterile water were used as control.

### 4.3 Gene isolation and bioinformatic analysis

Shuang You leaf cDNA was used for amplification of the coding sequences (CDSs) of *VaERF16* and *VaMYB306* with the primer pairs VaERF16_F/VaERF16_R and VaMYB306_F/VaMYB306_R, respectively (Table S1). Chromosomal localization was analysed using the Grape Genome Browser (https://www.genoscope.cns.fr/externe/GenomeBrowser/Vitis/). DNAMAN (Lynnon Biosoft) was used for sequence alignment. Construction of the phylogenetic tree and cluster analysis were performed using MEGA v. 10.1.8 (Pennsylvania State University). The SMART website (http://smart.embl‐heidelberg.de/) was used to determine the conserved protein domains. The three‐dimensional structure of VaERF16 was predicted using the SWISS‐MODEL website (https://swissmodel.expasy.org/).

### 4.4 Subcellular localization analysis

The *VaERF16* CDS (without stop codon) was inserted into the pEarleyGate101 vector for VaERF16‐YFP expression. The full‐length *VaMYB306* sequence without the stop codon was cloned into the pCAMBIA2300‐GFP vector for VaMYB306‐GFP expression.

*Agrobacterium tumefaciens* GV3101 containing the recombinant vectors was infiltrated into *N*. *benthamiana* leaves, which were then held at 22°C at 60% relative humidity under a 16‐h light/8‐h dark photoperiod for 2 days. The location of the nucleus was indicated by treatment with DAPI (4 μM) for 30 min before examination. YFP and GFP signals were detected by laser scanning confocal microscopy (FV1000MPE; Olympus).

### 4.5 Y2H assay

For the yeast transactivation assay, the full‐length *VaERF16* CDS was inserted into the pGBKT7 vector (Clontech). The VaERF16‐BD plasmid was transformed into Y2H Gold cells according to the manufacturer's instructions (Clontech), which were then cultured on three types of medium (SD/−Trp, SD/−Trp with 150 ng/ml AbA, and SD/−Trp with 150 ng/ml AbA and 50 μg/ml X‐α‐Gal) at 30°C for 2–3 days before observation. Combinations of AD/T with BD/p53 and BD/Lam were used as positive and negative controls, respectively. Methods for screening interacting proteins were based on the Matchmaker Gold Yeast Two‐Hybrid System (Clontech).

To test the interaction in yeast, the *VaERF16*D1 sequence was cloned into the pGBKT7 vector to express VaERF16D1‐BD, and the *VaMYB306* CDS was inserted into the pGADT7 vector to express VaMYB306‐AD. The two plasmids were then co‐transformed into Y2H Gold cells, and the positive strains were selected on SD/−Leu/−Trp/−Ade/−His medium containing 50 μg/ml X‐α‐Gal and 150 ng/ml AbA. Blue colouration indicates interaction between the two proteins.

### 4.6 BiFC analysis

The full‐length CDSs of *VaERF16* and *VaMYB306* (without their respective stop codons) were cloned into pSPYCE‐35S to express YFPNE‐VaERF16 and YFPNE‐VaMYB306 and into pSPYNE‐35S to express YFPNE‐VaERF16 and YFPNE‐VaMYB306. The pSPYNE‐VaERF16/VaMYB306‐pSPYCE and pSPYNE‐VaMYB306/VaERF16‐pSPYCE combinations, as well as the BiFC plasmids and negative controls, were transiently expressed in *N*. *benthamiana* leaves using *Agrobacterium*‐mediated transformation as previously described (Liu et al., 2010). After 24 h, the fluorescence signals were visualized using a confocal laser scanning microscope (TCS SP8; Leica). The specific primers used to make these constructs are listed in Table S1.

### 4.7 Co‐IP

For Co‐IP, *VaERF16* and *VaMYB306* CDSs were inserted into pCAMBIA2300‐GFP (to express VaERF16‐GFP) and pEarleyGate201 (to express VaMYB306‐HA). Cultures of *A. tumefaciens* EHA105 containing the *VaERF16*‐GFP/*VaMYB306*‐HA or GFP/*VaMYB306*‐HA plasmids were individually infiltrated into *N*. *benthamiana* leaves. Two days after infiltration, 0.4 g of flash‐frozen leaves was ground into a powder and homogenized in extraction buffer (1 M Tris‐HCl, pH 8, 10% SDS, 50% glycerol, 5% mercaptoethanol), before incubation with 3 μl anti‐HA (ABclonal) and 30 μl protein A/G PLUS‐Agarose: sc‐2003 (Santa Cruz Biotechnology, Inc.) overnight at 4°C with gentle shaking. The immune complexes were centrifuged at 2000 × *g* for 5 min and washed with extraction buffer. The supernatant was mixed with SDS‐PAGE sample loading buffer and subjected to western blot analysis as previously described (Yu et al., 2013). Mouse monoclonal anti‐GFP (TransGen Biotech) and anti‐HA antibodies (ABclonal) were used to detect the target proteins. IPKine horseradish peroxidase, goat anti‐mouse IgG HCS (A25112) (http://www.abbkine.com/) was used as a secondary antibody.

### 4.8 Split luciferase assay

The full‐length CDSs of *VaERF16* and *VaMYB306* (without their respective stop codons) were inserted into the pCB1300‐Cluc and pCB1300‐Nluc vectors, respectively. The plasmids were transferred to *A. tumefaciens* GV3101 and co‐infiltrated into 4‐week‐old *N. benthamiana* leaves. Two days after infiltration, the firefly luciferase substrate (0.3 mg/ml) was applied evenly on the back of the leaves, which were placed for 10 min in darkness. Luciferase imaging was performed using a charged‐coupled device camera (Andor; iKon‐M 934) and PlantLab software (BioImaging Solutions). Infiltrations with pCB1300‐*VaERF16*‐Cluc/pCB1300‐Nluc, pCB1300‐Nluc‐*VaMYB306*/pCB1300‐Cluc, and pCB1300‐Cluc/pCB1300‐Nluc were used as controls.

### 4.9 Yeast one‐hybrid assay

The Matchmaker Gold Yeast One‐Hybrid System (Clontech) was used for experimental analysis. The 1447‐bp *VaPDF1.2* promoter was amplified by PCR and inserted into the pABAi vector to generate pABAi‐pro*VaPDF1.2*. The vector was digested with *Bst*BI endonuclease (NEB) for linearization and transfected into the Y1H Gold yeast strain as a bait. The *VaERF16* and *VaMYB306* CDSs were cloned into pGADT7 to generate AD‐*VaERF16* and AD‐*VaMYB306*, respectively, as prey. The prey vector was separately transformed into the bait strains. Transformants were selected and grown on SD/−Leu medium with 200 ng/ml AbA to confirm positive interactions. pGADT7 + pABAi‐pro*VaPDF1.2* was used as the negative control.

### 4.10 Dual‑luciferase assays

The full‐length *VaERF16* and *VaMYB306* cDNAs were each separately cloned into the pGreenII 62‐SK transient expression vector to serve as effectors (62‐SK‐*VaERF16* and 62‐SK‐*VaMYB306*). The *VaPDF1.2* promoter was inserted into the pGreenII 0800‐LUC transient expression vector to serve as a reporter (pro*VaPDF1*.*2*‐LUC). All plasmids were individually transformed into *A. tumefaciens* GV3101. Tobacco leaves were co‐infiltrated with *A*. *tumefaciens* harbouring the reporter plasmid and different effector plasmids in the following combinations: pro*VaPDF1*.*2*‐LUC + 62‐SK‐*VaERF16*, pro*VaPDF1.2*‐LUC + 62‐SK‐*VaMYB306*, and pro*VaPDF1*.*2*‐LUC + 62‐SK‐*VaERF16* + 62‐SK‐*VaMYB306*. The empty pGreenII 62‐SK vector was used as a control, and the Dual Luciferase Reporter Gene Assay Kit (Beyotime) was used to measure the activities of firefly luciferase and Renilla luciferase with an Infinite M200 PRO enzyme labelling instrument (Tecan) as previously described (Gu et al., 2021). All experiments were carried out with three independent replicates.

### 4.11 *Agrobacterium*‐mediated transient expression in grape leaves and *B. cinerea* infection

The plasmid constructs, as well as an empty vector, were electroporated into *A. tumefaciens* GV3101. Cultures were incubated at 28°C in lysogeny broth liquid medium with shaking at 180 rpm for 16 h. After centrifugation at 8,200 × *g* for 10 min, the pelleted bacteria were resuspended in infiltration buffer (10 mM MES, pH 5.6, 10 mM MgCl_2_, 200 μM acetosyringone) to OD_600_ = 0.6. Detached grape leaves (leaves at nodes 3 and 4, counted from the top of vines) with similar ages and sizes were selected randomly, submerged in *A*. *tumefaciens* suspensions, and infiltrated for 30 min under a vacuum of 0.085 MPa. After vacuum treatment, the samples were placed in trays with the petioles wrapped in moist cotton for further analysis. Three independent biological repeats were conducted, and each biological repeat included at least 10 grape leaves.

For *B*. *cinerea* infection, the grape leaves were held for 2 days at 25°C in the dark and agar discs (containing uniform *B*. *cinerea* mycelia, diameter 0.5 cm) were placed on top of the grape leaves to infect, before incubation at 22°C under a 16‐h light/8‐h dark photoperiod at a relative humidity of 90%–100%. For trypan blue staining, leaves were submerged in trypan blue solution (20 ml ethanol, 10 ml phenol, 10 ml lactic acid, and 10 mg trypan blue dissolved in 10 ml sterile water). The tubes were subjected to a vacuum at 0.085 MPa for 30 min and then boiled for 5 min. The leaves were then bleached in 2.5 g/ml chloral hydrate solution for 24 h. *B*. *cinerea* conidia were observed using an automated fluorescence microscope (BX63; Olympus). For *B. cinerea* biomass measurement, three biological replicates were performed.

### 4.12 *A. thaliana* transformation and disease assays

*A. tumefaciens* GV3101 harbouring the 35S‐*VaERF16* plasmid was used for *A. thaliana* transformation as previously described (Wang et al., 2020). Three independent T_3_ transgenic lines were used for disease assays. *A. thaliana* leaves were infected with Pst DC3000 and *B*. *cinerea* following previously published methods (Whalen et al., 1991). Leaves were collected at 0, 24, 48, and 72 hpi for quantitative PCR (qPCR). Three days after Pst DC3000 inoculation, the leaves were used for measuring bacterial colonies (cfu/cm^2^) as previously described (Wang et al., 2020). *B*. *cinerea* biomass was determined in three biological replicates (primers are listed in Table S1). Callose deposition was analysed using an aniline blue assay, in which the leaves were decolourized with 95% ethanol and then stained with aniline blue solution for 24 h, before visualization using a fluorescence microscope (BX63; Olympus) with UV light. To observe cell death, 72 hpi leaves were submerged in trypan blue solution (20 ml ethanol, 10 ml phenol, 10 ml lactic acid, and 10 mg trypan blue dissolved in 10 ml sterile water) and boiled for 2 min. The stained leaves were bleached with 2.5 g/ml chloral hydrate solution. Leaves were collected 3 days after inoculation with Pst DC3000 and *B. cinerea* for DAB staining by immersion in a 1 mg/ml DAB solution (pH 3.8) for 8 h and then boiled in 95% ethanol for destaining. A commercial detection kit (Suzhou Keming Bioengineering Institute) was used to determine H_2_O_2_ content in *Arabidopsis* leaves as previously described (Moloi & van der Westhuizen, 2006).

### 4.13 Gene expression analysis by reverse transcription‐qPCR

Total RNA was extracted from grapes and *A. thaliana* using the Plant RNA Kit (Omega Bio‐tek) according to the manufacturer's instructions. cDNA was obtained using PrimeScript reverse transcriptase (TaKaRa Biotechnology). qPCR was carried out on a Step One Plus real‐time PCR System (Applied Biosystems) with SYBR Green, according to the user manual (TaKaRa Biotechnology). The specificity of primers was checked in the NCBI (https://www.ncbi.nlm.nih.gov/) database, using the Primer‐BLAST program. The validity and completeness of qPCR products were confirmed by agarose gel electrophoresis (Figure S9b). PCR amplification efficiency was predicted on the pcrEfficiency (http://srvgen.upct.es/efficiency.html) website (Figure S9a) (Mallona et al., 2011). Relative mRNA expression levels were calculated by the 2^−ΔΔ^ *^C^* ^t^ method, where ΔΔ*C*t = (*C*t_Target gene_ − *C*t_Actin_)_Time x_ − (*C*t_Target gene_ − *C*t_Actin_)_Time 0_ (Livak & Schmittgen, 2001). Grapevine *ACTIN7* (XM_002282480), *GAPDH* (XM_002278316.4), and *EF1‐α* (XM_002284888) and *A. thaliana Actin2* (AT3G18780), *EF1α* (AT5G60390), and *UBQ5* (AT3G62250) were used as internal reference genes. Data are presented as the mean (±*SD*) from three independent biological replicates. Specific primers used are listed in Table S1.

### 4.14 Statistical analysis

Statistical analysis was conducted using Student's two‐tailed *t* test (*p < 0.05, **p < 0.01). Data were generated from three biological repeats. Error bars indicate standard error of the mean.

## CONFLICT OF INTEREST

The authors declare no conflicts of interest.

## Supporting information


**FIGURE S1** Gene expression analysis of *ERF16* in grapevine. (a) Transcript levels of *ERF16* in fruits of *Vitis vinifera* ‘Red Globe’ (R.G.) and *Vitis amurensis* ‘Shuang You’ (S.Y.) after *Botrytis cinerea* inoculation. Inoculated and noninoculated are denoted as IN and CK, respectively. (b) The transcript levels of *VaERF16* in response to ethephon (Eth) treatment. (c) *ERF16* expression levels in different organs of Red Globe and Shuang You. (d) Reverse transcription‐quantitative PCR analysis of *VaERF16* in leaves treated with methyl jasmonate (MeJA). *ACTIN7* (XM_002282480), *GAPDH* (XM_002278316.4), and *EF1‐α* (XM_002284888) were used as internal reference genes. Results are shown as the means (±*SD*) of three biological repeats. Asterisks represent significant differences (*p < 0.05, **p < 0.01, Student’s two‐tailed *t* test) between inoculated and mock‐inoculated plants at the same time point

**FIGURE S2** *Botrytis cinerea* conidia development on transgenic *Arabidopsis thaliana* and wild‐type leaves. Leaves were harvested at 0, 24, 48, and 72 h postinoculation (hpi) to detect progression of *B. cinerea* colonization. Scale bar = 150 μm

**FIGURE S3** Overexpression of *VaERF16* in *Arabidopsis thaliana* improves resistance to *Pseudomonas syringae* pv. *tomato* (Pst) DC3000. (a) Disease symptoms on leaves of wild‐type (WT) and transgenic lines (L1, L2, and L3) after infection with Pst DC3000 for 72 h. Scale bar = 1 cm. (b) Bacterial colonies from WT and transgenic leaf samples were cultivated in Petri dishes. (c) Trypan blue detection of cell death after infection with Pst DC3000 for 72 h. 3,3′‐Diaminobenzidine (DAB) staining for H_2_O_2_ detection. Scale bar = 1 cm. (d) Detection of callose deposition in leaves at 24 h after Pst DC3000 inoculation using aniline blue staining. Scale bar = 150 μm. (e) Bacterial population assays in inoculated transgenic and WT leaves 72 h postinoculation. (f) Reverse transcription‐quantitative PCR analysis of defence‐related genes in *VaERF16*‐overexpressing (OE) lines and WT plants at 0, 24, 48, and 72 h after Pst DC3000 inoculation. *AtActin2* (AT3G18780), *EF1α* (AT5G60390), and *UBQ5* (AT3G62250) were used as internal reference genes. Results are shown as the means (±*SD*) of three biological assays. Statistical significance was determined with Student’s two‐tailed *t* test (*p < 0.05, **p < 0.01)

**FIGURE S4** Bioinformatic analysis of *VaMYB306*. (a) Amino acid sequence alignment of conserved motifs in VaMYB306 with homologues from other plant species. The R2 MYB domain is indicated with a red line, and the R3 domain is indicated with a light orange line. The sequences are from the following proteins: AtMYB306 (*Arabidopsis thaliana*, NP_190344.1), GhMYB306 (*Gossypium hirsutum*, XP_040970228.1), GmMYB306 (*Glycine max*, NP_001341097.1), MdMYB306 (*Malus domestica*, XP_028953285.1), MsMYB306 (*Medicago sativa*, AFJ53055.1), NtMYB306 (*Nicotiana tabacum*, XP_016432585), and SlMYB306 (*Solanum lycopersicum*, XP_004236011.1). (b) VaMYB306 phylogenetic analysis. VaMYB306 is indicated with a red circle. (c) Subcellular localization of VaMYB306 in the epidermal cells of tobacco leaves. VaMYB306‐GFP fusion proteins were observed using a confocal microscope. Scale bar = 33.2 μm. (d) Tissue‐specific *MYB306* gene expression profiles in Red Globe and Shuang You

**FIGURE S5** Gene expression profiles of *MYB306* in grapevine. (a,c) Transcript levels of *MYB306* after mock treatment and *Botrytis cinerea* infection in *Vitis vinifera* ’Red Globe’ and *Vitis amurensis* ’Shuang You’ fruits and leaves. The fruits were sampled 0, 1, 3, and 5 days after inoculation. The leaves were collected 4, 8, 18, and 36 h after treatment. Inoculated and noninoculated are denoted as IN and CK, respectively. (b,d) Transcript levels of *MYB306* in response to different hormone treatments. Shuang You leaves were treated with 0.5 g/L ethephon and 50 μM methyl jasmonate (MeJA). Mock, control leaves treated with distilled water. *ACTIN7* (XM_002282480), *GAPDH* (XM_002278316.4), and *EF1‐α* (XM_002284888) were used as internal reference genes. Results are indicated as mean values from three biological replicates. Error bars indicate *SD*. Statistical significance was determined by Student’s two‐tailed *t* test (*p < 0.05, **p < 0.01)

**FIGURE S6** Transient overexpression of *VaERF16* or *VaMYB306* in leaves of *Vitis vinifera* ’Thompson Seedless’ enhances resistance to *Botrytis cinerea*. (a–d) Phenotype of infiltrated leaves (wild type [WT], empty overexpression [OE] vector, *VaERF16* OE, and *VaMYB306* OE) after inoculation with *B. cinerea*. Scale bar = 1 cm. Each row of photographs represents an independent experiment. (e) Trypan blue staining to visualize the development of *B. cinerea* conidia. Scale bar = 150 μm. The leaves were collected at 24, 48, and 72 h after inoculation. (f) Quantitative PCR quantification of *B. cinerea* colonization. Total genomic DNA from *B. cinerea*‐infected leaves was isolated at 0, 24, 48, and 72 h after inoculation. *B. cinerea Actin* was used to determine *B. cinerea* biomass in infected plant tissues. (g) Reverse transcription‐quantitative PCR analysis of *VaERF16* and *VaMYB306* in infiltrated leaves. Asterisks represent significant differences between infiltrated leaves (*VaERF16* OE, *VaMYB306* OE) and WT leaves. (h) Expression profiles of defence‐related genes *PDF1.2* and *ERF20* in infiltrated leaves at 0, 24, 48, and 72 h after *B. cinerea* inoculation. *ACTIN7* (XM_002282480), *GAPDH* (XM_002278316.4), and *EF1‐α* (XM_002284888) were used as internal reference genes. Error bars indicate the *SD* from three independent experiments. Asterisks represent significant differences (*p < 0.05, **p < 0.01, Student’s two‐tailed *t* test)

**FIGURE S7** Transient silencing of *VaERF16* and *VaMYB306* in leaves of *Vitis quinquangularis* ’Ju Meigui’ reduces resistance to *Botrytis cinerea*. (a–c) The disease symptoms of infiltrated leaves (wild type [WT], *VaERF16*‐RNAi and *VaMYB306*‐RNAi) after *B. cinerea* inoculation. The leaves were collected 0, 24, 48, and 72 h postinoculation. Scale bar = 1 cm. Each row of photographs represents an independent experiment. (d) Trypan blue staining was performed to detect the development of *B. cinerea* conidia. Scale bar = 150 μm. (e) Quantitative PCR quantification of *B. cinerea* colonization. Total genomic DNA from *B. cinerea*‐infected leaves was isolated at 0, 24, 48, and 72 h after inoculation. *B. cinerea Actin* was used to determine *B. cinerea* biomass in infected plant tissues. (f) Gene expression analysis of the defence‐related genes *PDF1.2* and *ERF20* in infiltrated leaves 0, 24, 48, and 72 h after *B. cinerea* inoculation. *ACTIN7* (XM_002282480), *GAPDH* (XM_002278316.4), and *EF1‐α* (XM_002284888) were used as internal reference genes. Error bars indicate the *SD* from three independent experiments. Asterisks represent significant differences (*p < 0.05, **p < 0.01, Student’s two‐tailed *t* test)

**FIGURE S8** Expression profiles of *VaERF16* and *VaMYB306* in infiltrated leaves of (a) *Vitis amurensis* ’Shuang You’ and (b) *Vitis quinquangularis* ’Ju Meigui’. Asterisks represent significant differences (*p < 0.05, **p < 0.01, Student’s two‐tailed *t* test) between leaves infiltrated with *Agrobacterium* containing *VaERF16*‐RNAi or *VaMYB306*‐RNAi vectors or negative control

**FIGURE S9** PCR amplification efficiency prediction and quality testing of primers used in this study. (a) PcrEfficiency (http://srvgen.upct.es/efficiency.html) was used for PCR amplification efficiency prediction. (b) The quality of primers was tested by agarose gel electrophoresis. The numbers indicate the specific primers. Marker: Trans DNA marker Ⅱ

**TABLE S1** Primers used in this study

## Data Availability

All data supporting the findings of this study are available within the paper and its supplementary data published online.
