# Supplementary material for: The transcription factors VaERF16 and VaMYB306 interact to enhance resistance of grapevine to Botrytis cinerea infection
Source: Mol Plant Pathol. 2022 Jul 12;23(10):1415–32. doi: 10.1111/mpp.13223 (PMC9452770; doi:10.1111/mpp.13223)
Supplement: Supplementary file 1 — FIGURE S1 Gene expression analysis of ERF16 in grapevine. (a) Transcript levels of ERF16 in fruits of Vitis vinifera ‘Red Globe’ (R.G.) and Vitis amurensis ‘Shuang You’ (S.Y.) after Botrytis cinerea inoculation. Inoculated and noninoculated are denoted as IN and CK, respectively. (b) The transcript levels of VaERF16 in response to ethephon (Eth) treatment. (c) ERF16 expression levels in different organs of Red Globe and Shuang You. (d) Reverse transcription‐quantitative PCR analysis of VaERF16 in leaves treated with methyl jasmonate (MeJA). ACTIN7 (XM_002282480), GAPDH (XM_002278316.4), and EF1‐α (XM_002284888) were used as internal reference genes. Results are shown as the means (±SD) of three biological repeats. Asterisks represent significant differences (*p < 0.05, **p < 0.01, Student’s two‐tailed t test) between inoculated and mock‐inoculated plants at the same time point [file MPP-23-1415-s007.docx]

**
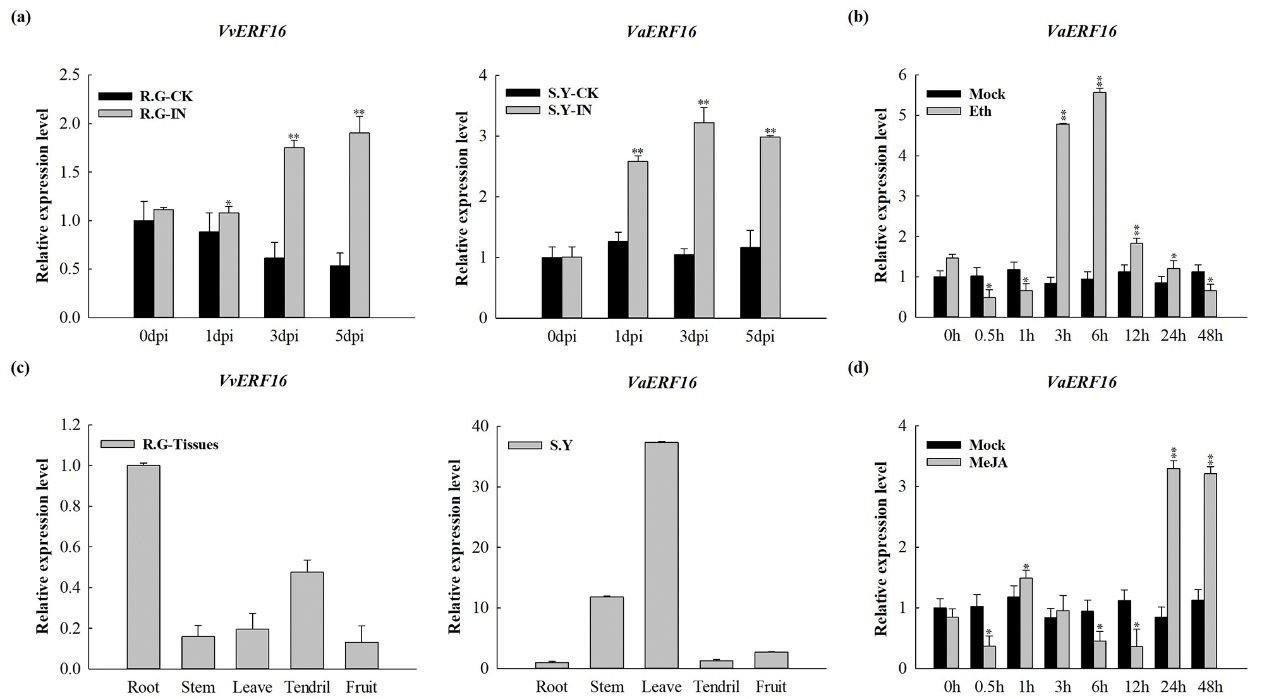
**

**Figure S1** Transcriptional levels analysis of *ERF16* in grapevine. (a) Transcriptional levels of *ERF16* in fruits of *Vitis vinifera* “Red Globe” (R.G) and *V. amurensis* “Shuang you” (S.Y) after *Botrytis cinerea* inoculation. ‘inoculated’ and ‘non-inoculated’ are denoted as ‘IN’ and ‘CK’, respectively. (b) The transcriptional levels of *VaERF16* in response to ethrel (Eth) treatment. (c) *ERF16* expression profiles in different organs of “Red Globe” (R.G) and “Shuang you” (S.Y). (d) qRT-PCR analysis at the transcriptional levels of *VaERF16* in leaves treated with methyl jasmonate (MeJA). *ACTIN7* (XM_002282480), *GAPDH* (XM_002278316.4) and *EF1-α* (XM_002284888) were used as internal reference genes. Results are shown as the means (±SD) of three biological repeats. Asterisks represent significant differences (**P*< 0.05, ***P*< 0.01, Student's two-tailed *t* test) between inoculated and mock-inoculated plants at the same time point.
