# Supplementary material for: The transcription factors VaERF16 and VaMYB306 interact to enhance resistance of grapevine to Botrytis cinerea infection
Source: Mol Plant Pathol. 2022 Jul 12;23(10):1415–32. doi: 10.1111/mpp.13223 (PMC9452770; doi:10.1111/mpp.13223)
Supplement: Supplementary file 2 — FIGURE S2 Botrytis cinerea conidia development on transgenic Arabidopsis thaliana and wild‐type leaves. Leaves were harvested at 0, 24, 48, and 72 h postinoculation (hpi) to detect progression of B. cinerea colonization. Scale bar = 150 μm [file MPP-23-1415-s010.docx]

**
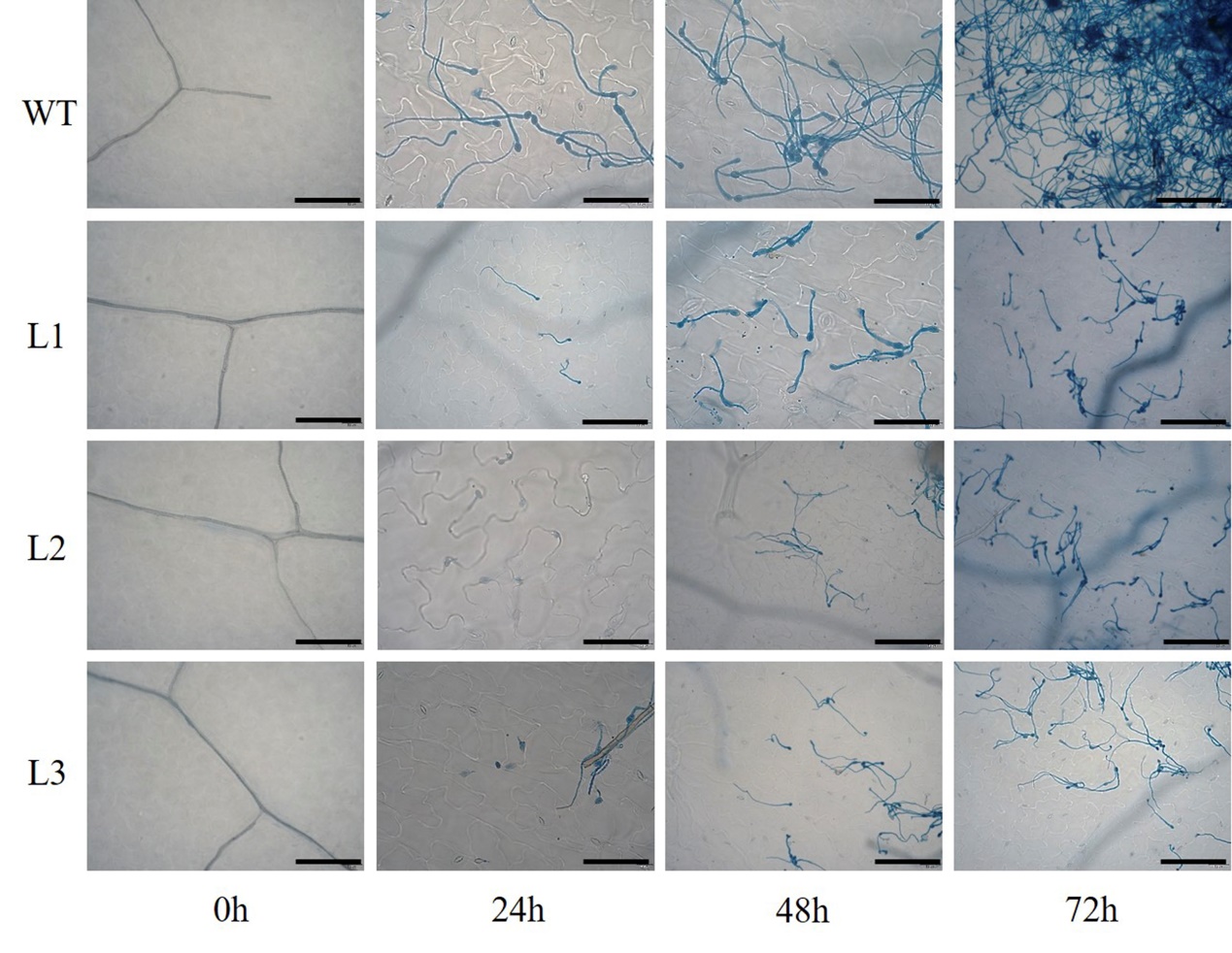
**

**Figure S2** *B. cinerea* conidia development on transgenic *A. thaliana* and WT leaves. Leaves were harvested at 0, 24, 48, 72 hours post inoculation (hpi) to detect progression of *B. cinerea* colonization. Scale bar = 150 μm.
