# Supplementary material for: The transcription factors VaERF16 and VaMYB306 interact to enhance resistance of grapevine to Botrytis cinerea infection
Source: Mol Plant Pathol. 2022 Jul 12;23(10):1415–32. doi: 10.1111/mpp.13223 (PMC9452770; doi:10.1111/mpp.13223)
Supplement: Supplementary file 3 — FIGURE S3 Overexpression of VaERF16 in Arabidopsis thaliana improves resistance to Pseudomonas syringae pv. tomato (Pst) DC3000. (a) Disease symptoms on leaves of wild‐type (WT) and transgenic lines (L1, L2, and L3) after infection with Pst DC3000 for 72 h. Scale bar = 1 cm. (b) Bacterial colonies from WT and transgenic leaf samples were cultivated in Petri dishes. (c) Trypan blue detection of cell death after infection with Pst DC3000 for 72 h. 3,3′‐Diaminobenzidine (DAB) staining for H2O2 detection. Scale bar = 1 cm. (d) Detection of callose deposition in leaves at 24 h after Pst DC3000 inoculation using aniline blue staining. Scale bar = 150 μm. (e) Bacterial population assays in inoculated transgenic and WT leaves 72 h postinoculation. (f) Reverse transcription‐quantitative PCR analysis of defence‐related genes in VaERF16‐overexpressing (OE) lines and WT plants at 0, 24, 48, and 72 h after Pst DC3000 inoculation. AtActin2 (AT3G18780), EF1α (AT5G60390), and UBQ5 (AT3G62250) were used as internal reference genes. Results are shown as the means (±SD) of three biological assays. Statistical significance was determined with Student’s two‐tailed t test (*p < 0.05, **p < 0.01) [file MPP-23-1415-s004.docx]

**
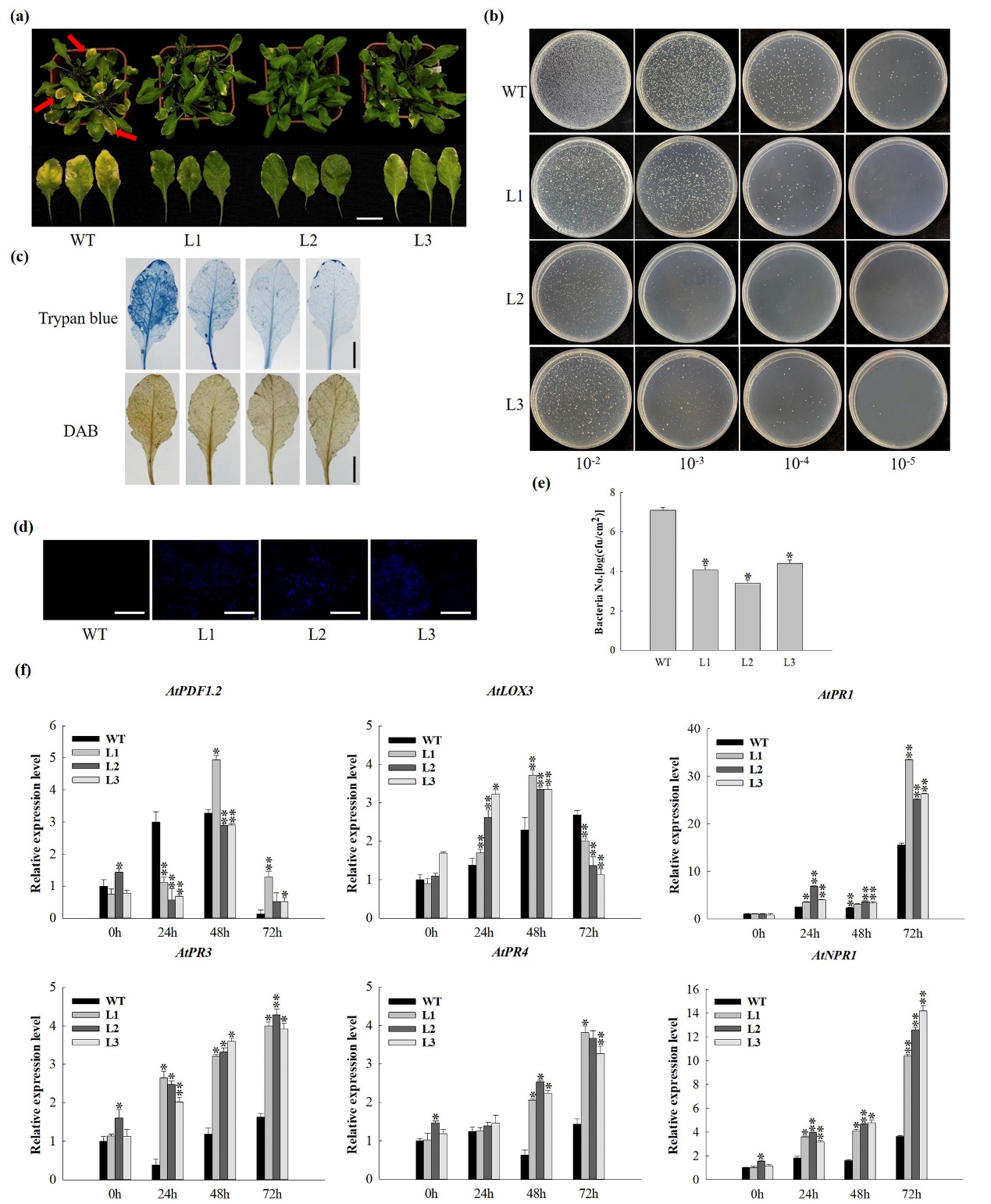
**

**Figure S3** Overexpression of *VaERF16* in *A. thaliana* improves resistance to *Pst DC3000.* (a) Disease symptoms on leaves of wild type (WT) and transgenic lines (L1, L2 and L3) after infection with *Pst DC3000* for 72 h. Scale bar = 1 cm. (b) Bacterial colonies from WT and transgenic line leaf samples were cultivated in Petri dishes. (c) Trypan blue detection of cell death after infection with *Pst DC3000* for 72 h. 3,3'-diaminobenzidine (DAB) staining for H_2_O_2_ accumulation. Scale bar = 1 cm. (d) Detection of callose deposition in leaves at 24 h after *Pst DC3000* inoculation using aniline blue staining. Scale bars = 150 μm. (e) Bacterial population assays in inoculated transgenic and WT leaves 72 hours post-inoculation. (f) qRT-PCR analysis at the transcriptional levels of defense-related genes in *VaERF16* over-expressing OE lines and WT plants at 0, 24, 48 and 72 h after *Pst DC3000* inoculation. *AtActin2* (AT3G18780), *EF1α* (AT5G60390) and *UBQ5* (AT3G62250) were used as internal reference genes. Results are shown as the means (±SD) of three biological assays. Statistical significance was determined with a Student’s two-tailed t test (*, *P* < 0.05; **, *P* < 0.01).
