# Supplementary material for: The transcription factors VaERF16 and VaMYB306 interact to enhance resistance of grapevine to Botrytis cinerea infection
Source: Mol Plant Pathol. 2022 Jul 12;23(10):1415–32. doi: 10.1111/mpp.13223 (PMC9452770; doi:10.1111/mpp.13223)
Supplement: Supplementary file 4 — FIGURE S4 Bioinformatic analysis of VaMYB306. (a) Amino acid sequence alignment of conserved motifs in VaMYB306 with homologues from other plant species. The R2 MYB domain is indicated with a red line, and the R3 domain is indicated with a light orange line. The sequences are from the following proteins: AtMYB306 (Arabidopsis thaliana, NP_190344.1), GhMYB306 (Gossypium hirsutum, XP_040970228.1), GmMYB306 (Glycine max, NP_001341097.1), MdMYB306 (Malus domestica, XP_028953285.1), MsMYB306 (Medicago sativa, AFJ53055.1), NtMYB306 (Nicotiana tabacum, XP_016432585), and SlMYB306 (Solanum lycopersicum, XP_004236011.1). (b) VaMYB306 phylogenetic analysis. VaMYB306 is indicated with a red circle. (c) Subcellular localization of VaMYB306 in the epidermal cells of tobacco leaves. VaMYB306‐GFP fusion proteins were observed using a confocal microscope. Scale bar = 33.2 μm. (d) Tissue‐specific MYB306 gene expression profiles in Red Globe and Shuang You [file MPP-23-1415-s009.docx]

**
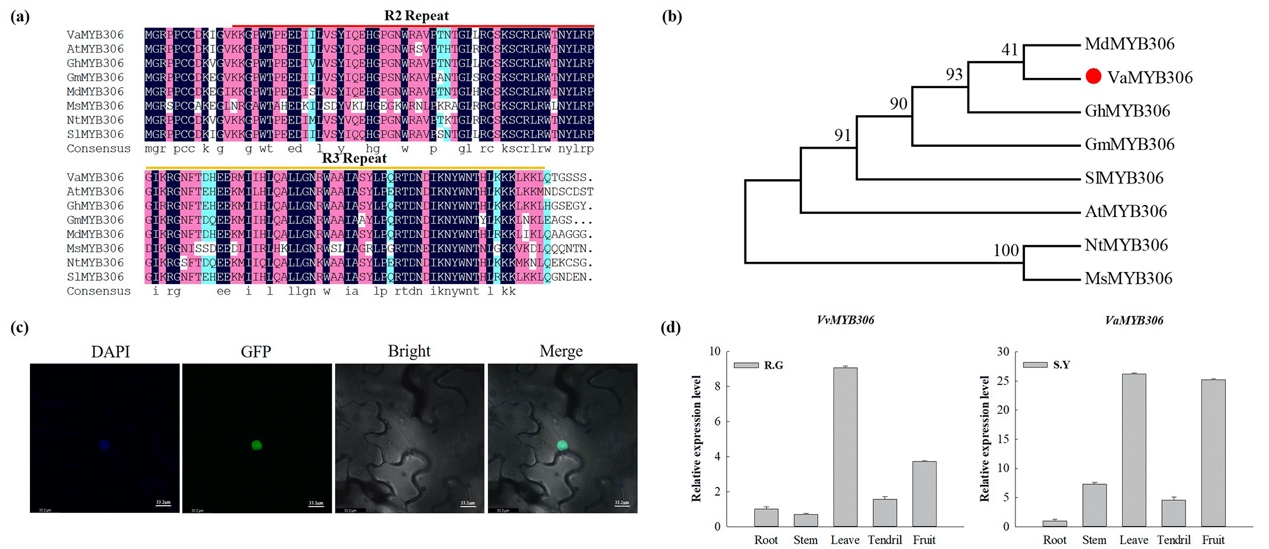
**

**Figure S4** Bioinformatic analysis of *VaMYB306*. (a) Conserved VaMYB306 motifs in an alignment with homologs from other plant species. The R2 MYB domain is indicated with a red line, and the R3 domain with a light orange line. The sequences are from the following: AtMYB306 (*Arabidopsis thaliana*, NP_190344.1), GhMYB306 (*Gossypium hirsutum*, XP_040970228.1), GmMYB306 (*Glycine max*, NP_001341097.1), MdMYB306 (*Malus domestica*, XP_028953285.1), MsMYB306 (*Medicago sativa*, AFJ53055.1), NtMYB306 (*Nicotiana tabacum*, XP_016432585), and SlMYB306 (*Solanum lycopersicum*, XP_004236011.1). (b) VaMYB306 phylogenetic analysis. VaMYB306 is indicated with a red circle. (c) Subcellular localization of VaMYB306 in the epidermal cells of tobacco leaves. VaMYB306-GFP fusion proteins were observed using a confocal microscope. Scale bars = 33.2 μm. (d) Tissue-specific *MYB306* expression profiles in “Red Globe” (R.G) and “Shuang you” (S.Y).
