# Supplementary material for: The transcription factors VaERF16 and VaMYB306 interact to enhance resistance of grapevine to Botrytis cinerea infection
Source: Mol Plant Pathol. 2022 Jul 12;23(10):1415–32. doi: 10.1111/mpp.13223 (PMC9452770; doi:10.1111/mpp.13223)
Supplement: Supplementary file 5 — FIGURE S5 Gene expression profiles of MYB306 in grapevine. (a,c) Transcript levels of MYB306 after mock treatment and Botrytis cinerea infection in Vitis vinifera ’Red Globe’ and Vitis amurensis ’Shuang You’ fruits and leaves. The fruits were sampled 0, 1, 3, and 5 days after inoculation. The leaves were collected 4, 8, 18, and 36 h after treatment. Inoculated and noninoculated are denoted as IN and CK, respectively. (b,d) Transcript levels of MYB306 in response to different hormone treatments. Shuang You leaves were treated with 0.5 g/L ethephon and 50 μM methyl jasmonate (MeJA). Mock, control leaves treated with distilled water. ACTIN7 (XM_002282480), GAPDH (XM_002278316.4), and EF1‐α (XM_002284888) were used as internal reference genes. Results are indicated as mean values from three biological replicates. Error bars indicate SD. Statistical significance was determined by Student’s two‐tailed t test (*p < 0.05, **p < 0.01) [file MPP-23-1415-s002.docx]

**
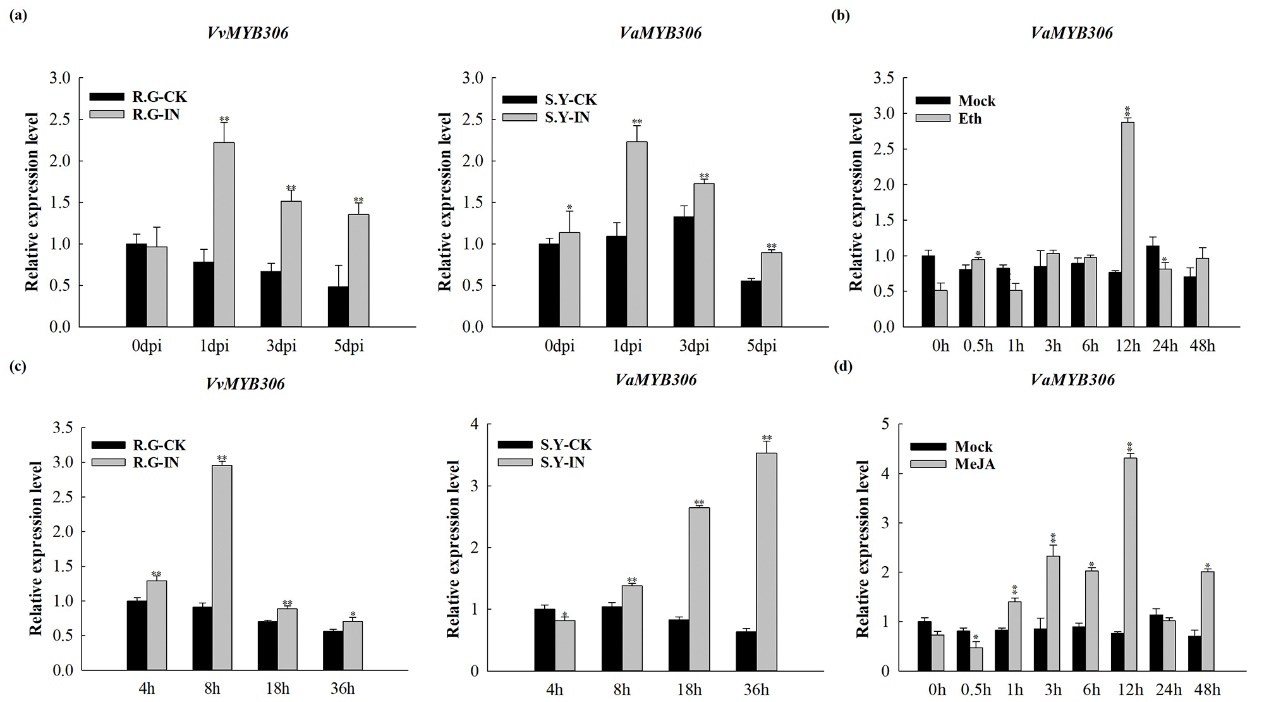
**

**Figure S5** Expression profiles of *MYB306* in grapevine. (a,c) Transcriptional levels of *MYB306* after mock treatment and *B. cinerea* infection in R.G and S.Y fruits and leaves. The fruits were sampled 0, 1, 3 and 5 days after inoculation. The leaves were collected 4, 8, 18 and 36 h after treatment. ‘Inoculated’ and ‘non-inoculated’ are denoted as ‘IN’ and ‘CK’, respectively. (b,d) Transcriptional levels of *MYB306* in response to different hormone treatments. S.Y leaves were treated with 0.5 g/L ethrel and 50 μM methyl jasmonate (MeJA). Mock, control leaves treated with distilled water. *ACTIN7* (XM_002282480), *GAPDH* (XM_002278316.4) and *EF1-α* (XM_002284888) were used as internal reference genes. Results are indicated as mean values from three biological replicates. Error bars indicate SD. Statistical significance was determined by a Student’s two-tailed t test (*, *P* < 0.05; **, *P* < 0.01).
