# Supplementary material for: The transcription factors VaERF16 and VaMYB306 interact to enhance resistance of grapevine to Botrytis cinerea infection
Source: Mol Plant Pathol. 2022 Jul 12;23(10):1415–32. doi: 10.1111/mpp.13223 (PMC9452770; doi:10.1111/mpp.13223)
Supplement: Supplementary file 6 — FIGURE S6 Transient overexpression of VaERF16 or VaMYB306 in leaves of Vitis vinifera ’Thompson Seedless’ enhances resistance to Botrytis cinerea. (a–d) Phenotype of infiltrated leaves (wild type [WT], empty overexpression [OE] vector, VaERF16 OE, and VaMYB306 OE) after inoculation with B. cinerea. Scale bar = 1 cm. Each row of photographs represents an independent experiment. (e) Trypan blue staining to visualize the development of B. cinerea conidia. Scale bar = 150 μm. The leaves were collected at 24, 48, and 72 h after inoculation. (f) Quantitative PCR quantification of B. cinerea colonization. Total genomic DNA from B. cinerea‐infected leaves was isolated at 0, 24, 48, and 72 h after inoculation. B. cinerea Actin was used to determine B. cinerea biomass in infected plant tissues. (g) Reverse transcription‐quantitative PCR analysis of VaERF16 and VaMYB306 in infiltrated leaves. Asterisks represent significant differences between infiltrated leaves (VaERF16 OE, VaMYB306 OE) and WT leaves. (h) Expression profiles of defence‐related genes PDF1.2 and ERF20 in infiltrated leaves at 0, 24, 48, and 72 h after B. cinerea inoculation. ACTIN7 (XM_002282480), GAPDH (XM_002278316.4), and EF1‐α (XM_002284888) were used as internal reference genes. Error bars indicate the SD from three independent experiments. Asterisks represent significant differences (*p < 0.05, **p < 0.01, Student’s two‐tailed t test) [file MPP-23-1415-s005.docx]

**
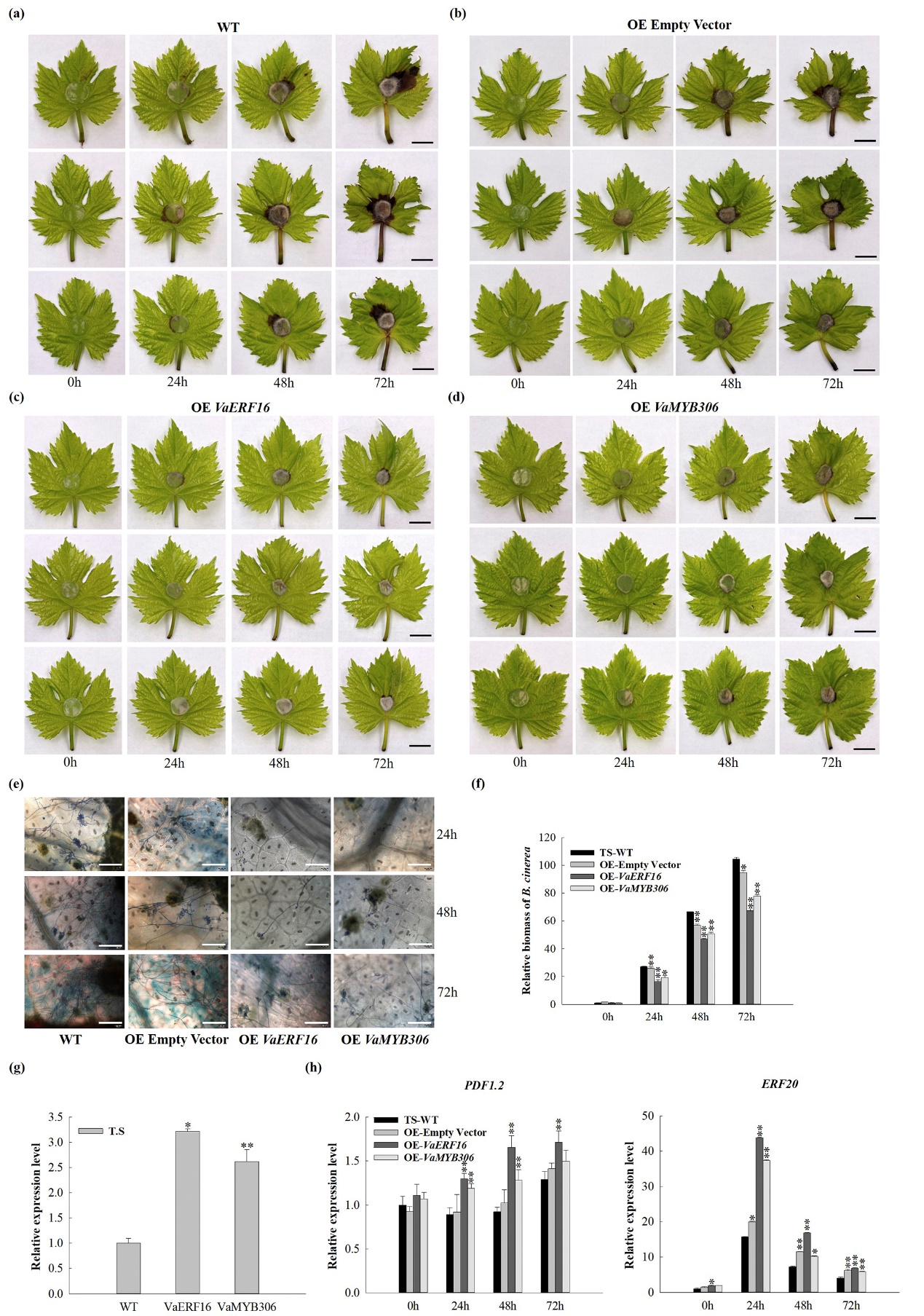
**

**Figure S6** Transient overexpression of *VaERF16* or *VaMYB306* in leaves of “Thompson Seedless” enhances resistance to *Botrytis cinerea.* (a-d) Phenotype of infiltrated leaves (WT, OE empty vector, OE *VaERF16* and OE *VaERF306*) after inoculation with *B. cinerea* (Scale bars = 1 cm). Each row of photos represents an independent experiment. (e) Trypan blue staining detecting the development of *B. cinerea* conidia (Scale bars = 150 μm). The leaves were collected at 24, 48 and 72 h after inoculation. (f) qRT-PCR quantification of *B. cinerea* colonization. Total genomic DNA from *B. cinerea*-infected leaves was isolated at 0, 24, 48 and 72 h after inoculation. *B. Cinerea Actin* was used to determine *B. cinerea* biomass in infected plant tissues. (g) Transcriptional levels analysis of *VaERF16* and *VaMYB306* in infiltrated leaves determined by quantitative real time (qRT)-PCR. Asterisks represent significant differences between infiltrated leaves (OE *VaERF16*, OE *VaMYB306*) and WT leaves. (h) Expression profiles of defense-related genes *PDF1.2* and *ERF20* in infiltrated leaves at 0, 24, 48 and 72 h after *B. cinerea* inoculation. *ACTIN7* (XM_002282480), *GAPDH* (XM_002278316.4) and *EF1-α* (XM_002284888) were used as internal reference genes. Error bars indicate the SD from three independent experiments. Asterisks represent significant differences (**P*< 0.05, ***P*< 0.01, Student's two-tailed *t* test).
