# Supplementary material for: The transcription factors VaERF16 and VaMYB306 interact to enhance resistance of grapevine to Botrytis cinerea infection
Source: Mol Plant Pathol. 2022 Jul 12;23(10):1415–32. doi: 10.1111/mpp.13223 (PMC9452770; doi:10.1111/mpp.13223)
Supplement: Supplementary file 7 — FIGURE S7 Transient silencing of VaERF16 and VaMYB306 in leaves of Vitis quinquangularis ’Ju Meigui’ reduces resistance to Botrytis cinerea. (a–c) The disease symptoms of infiltrated leaves (wild type [WT], VaERF16‐RNAi and VaMYB306‐RNAi) after B. cinerea inoculation. The leaves were collected 0, 24, 48, and 72 h postinoculation. Scale bar = 1 cm. Each row of photographs represents an independent experiment. (d) Trypan blue staining was performed to detect the development of B. cinerea conidia. Scale bar = 150 μm. (e) Quantitative PCR quantification of B. cinerea colonization. Total genomic DNA from B. cinerea‐infected leaves was isolated at 0, 24, 48, and 72 h after inoculation. B. cinerea Actin was used to determine B. cinerea biomass in infected plant tissues. (f) Gene expression analysis of the defence‐related genes PDF1.2 and ERF20 in infiltrated leaves 0, 24, 48, and 72 h after B. cinerea inoculation. ACTIN7 (XM_002282480), GAPDH (XM_002278316.4), and EF1‐α (XM_002284888) were used as internal reference genes. Error bars indicate the SD from three independent experiments. Asterisks represent significant differences (*p < 0.05, **p < 0.01, Student’s two‐tailed t test) [file MPP-23-1415-s008.docx]

**
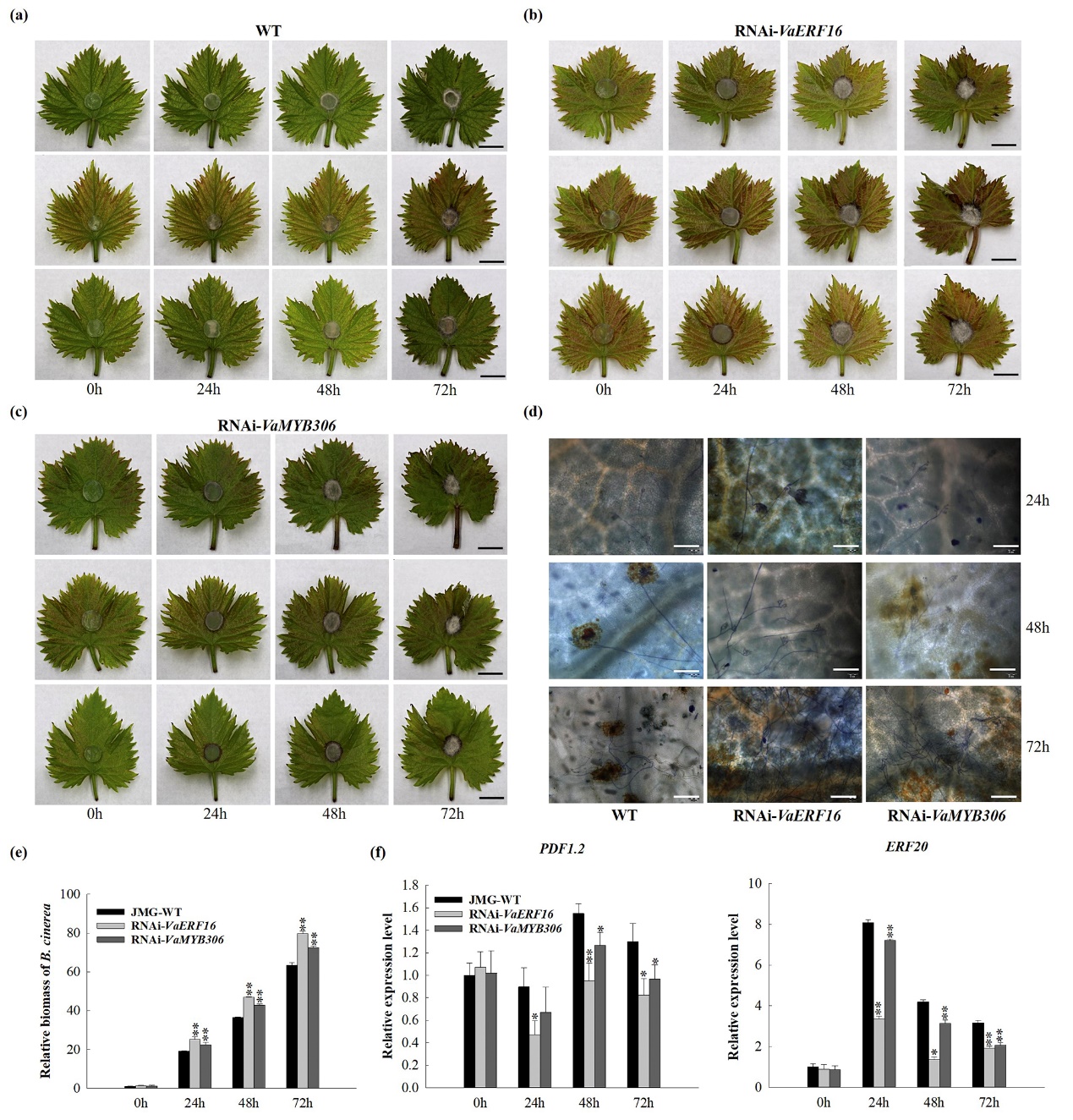
**

**Figure S7** Transient silencing of *VaERF16* and *VaMYB306* in leaves of “Ju meigui” reduces resistance to *Botrytis cinerea.* (a-c) The disease symptoms of infiltrated leaves (WT, RNAi-*VaERF16* and RNAi-*VaERF306*) after *B. cinerea* inoculation. The leaves were collected 0, 24, 48 and 72 hours post inoculation (Scale bars = 1 cm). Each row of photos represents an independent experiment. (d) Trypan blue staining was performed to detect the development of *Botrytis cinerea* conidia (Scale bars = 150 μm). (e) qRT-PCR quantification of *B. cinerea* colonization. Total genomic DNA from *B. cinerea*-infected leaves was isolated at 0, 24, 48 and 72 h after inoculation. *B. Cinerea Actin* was used to determine *B. cinerea* biomass in infected plant tissues. (f) Transcriptional levels analysis of defense-related genes *PDF1.2* and *ERF20* in infiltrated leaves after *B. cinerea* inoculation 0, 24, 48 and 72 h. *ACTIN7* (XM_002282480), *GAPDH* (XM_002278316.4) and *EF1-α* (XM_002284888) were used as internal reference genes. Error bars indicate the SD from three independent experiments. Asterisks represent significant differences (**P* < 0.05, ***P*< 0.01, Student's two-tailed *t* test).
