# Supplementary material for: The transcription factors VaERF16 and VaMYB306 interact to enhance resistance of grapevine to Botrytis cinerea infection
Source: Mol Plant Pathol. 2022 Jul 12;23(10):1415–32. doi: 10.1111/mpp.13223 (PMC9452770; doi:10.1111/mpp.13223)
Supplement: Supplementary file 8 — FIGURE S8 Expression profiles of VaERF16 and VaMYB306 in infiltrated leaves of (a) Vitis amurensis ’Shuang You’ and (b) Vitis quinquangularis ’Ju Meigui’. Asterisks represent significant differences (*p < 0.05, **p < 0.01, Student’s two‐tailed t test) between leaves infiltrated with Agrobacterium containing VaERF16‐RNAi or VaMYB306‐RNAi vectors or negative control [file MPP-23-1415-s003.docx]

**
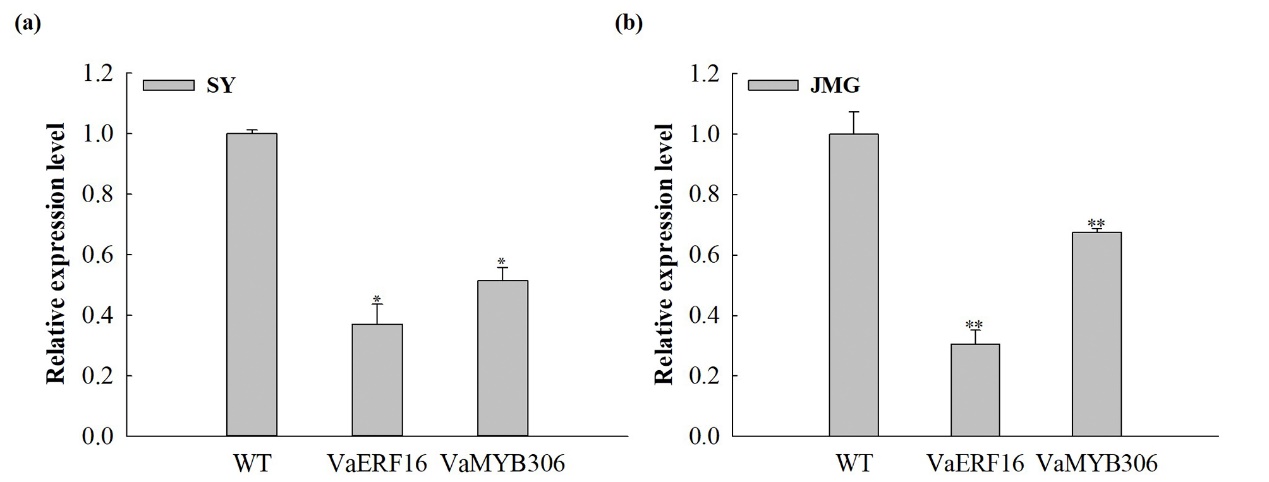
**

**Figure S8** Expression profiles of *VaERF16* and *VaMYB306* in infiltrated leaves of (a) “Shuang you” and (b) “Ju meigui”. Asterisks represent significant differences (**P* < 0.05, ***P*< 0.01, Student's two-tailed *t* test) between leaves infiltrated with *Agrobacterium* containing RNAi-*VaERF16* or RNAi-*VaMYB306* and Agrobacterium alone.
