# Supplementary material for: The transcription factors VaERF16 and VaMYB306 interact to enhance resistance of grapevine to Botrytis cinerea infection
Source: Mol Plant Pathol. 2022 Jul 12;23(10):1415–32. doi: 10.1111/mpp.13223 (PMC9452770; doi:10.1111/mpp.13223)
Supplement: Supplementary file 9 — FIGURE S9 PCR amplification efficiency prediction and quality testing of primers used in this study. (a) PcrEfficiency (http://srvgen.upct.es/efficiency.html) was used for PCR amplification efficiency prediction. (b) The quality of primers was tested by agarose gel electrophoresis. The numbers indicate the specific primers. Marker: Trans DNA marker Ⅱ [file MPP-23-1415-s001.docx]

**
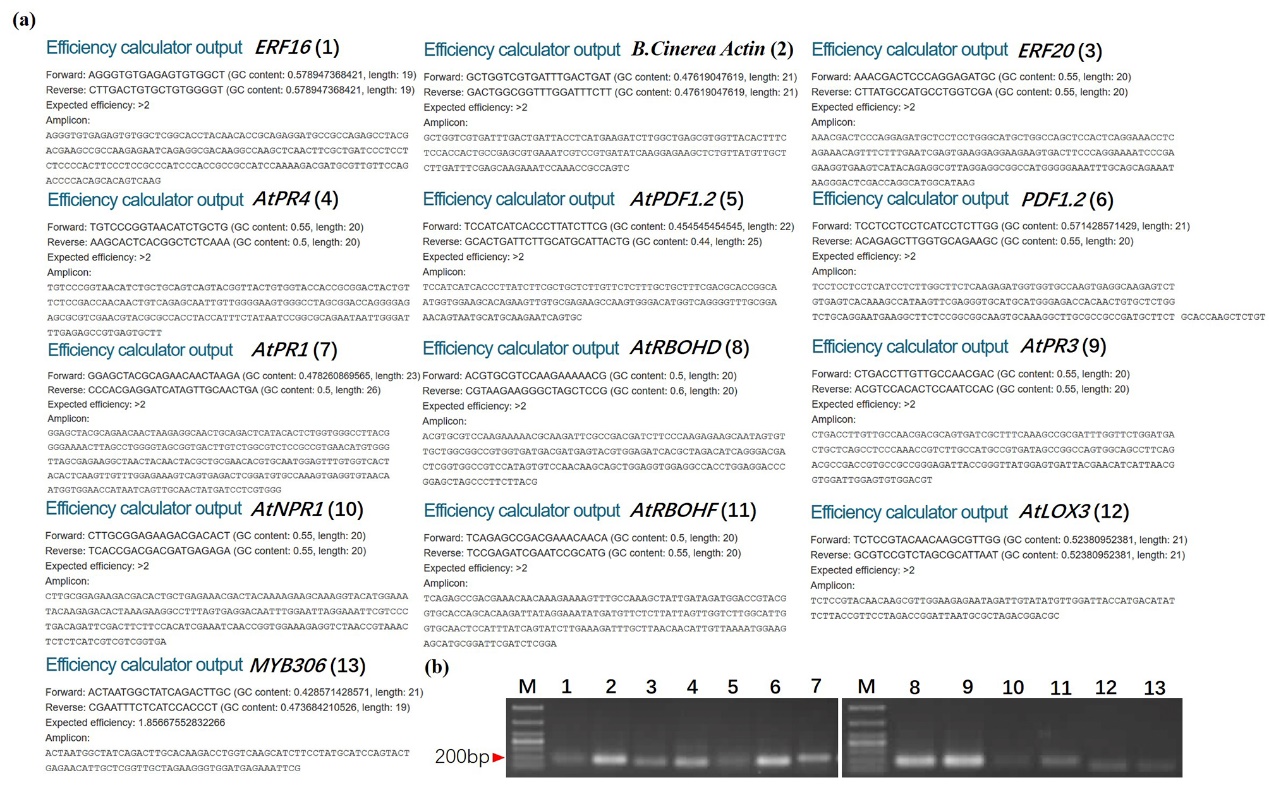
**

**Figure S9** PCR amplification efficiency prediction and quality testing of primers used in this study. (a) PcrEfficiency (<http://srvgen.upct.es/efficiency.html>) was used for PCR amplification efficiency prediction. (b) The quality of primers was tested by Agarose gel electrophoresis. The numbers above represent the specific primer. Marker: Trans DNA marker Ⅱ.
