# Supplementary material for: The transcription factors VaERF16 and VaMYB306 interact to enhance resistance of grapevine to Botrytis cinerea infection
Source: Mol Plant Pathol. 2022 Jul 12;23(10):1415–32. doi: 10.1111/mpp.13223 (PMC9452770; doi:10.1111/mpp.13223)
Supplement: Supplementary file 10 — TABLE S1 Primers used in this study [file MPP-23-1415-s006.docx]

| **Table S1 Primers used in this study.** | | | |
| --- | --- | --- | --- |
| **Primer name** | **Accession number** | **Forward sequence** | **Reverse sequence** |
| **Primers used for gene clone and vector construction** | | | |
| *VaERF16* Clone | CBI22960.3 | ATGTGTGGAGGTGCAATTATATCCG | TCAAAACGCAGCGCTGCATACATGC |
| *VaMYB306* Clone | XP_002283575 | ATGGGAAGACCACCTTGCTGTGATA | TCAGAAAAAATCAGGACTTTCATCT |
| pEarleyGate101-*VaERF16* |  | ggggacaagtttgtacaaaaaagcaggctgcATGTGTGGAGGTGCAATT | ggggaccactttgtacaagaaagctgggtcAAACGCAGCGCTGCATAC |
| *VaMYB306*-GFP |  | ggggacgagctcaacggtaccATGGGAAGACCACCTTGCTGT | catggttttgtcgactctagaGAAAAAATCAGGACTTTCATCTAATGA |
| pGBKT7-*VaERF16* |  | CGGAATTCATGTGTGGAGGTGCAATTATATCCG EcoRI | AACTGCAGTCAAAACGCAGCGCTGCATACATGC PstI |
| pGBKT7-*VaERF16* D1 |  | CGGAATTCATGTGTGGAGGTGCAATTATATCCG EcoRI | CGGGATCCATAGGTCATTAATGGTGGTGGTGGT BamHI |
| pGBKT7-*VaERF16* D2 |  | CGGAATTCATGTGTGGAGGTGCAATTATATCCG EcoRI | CGGGATCCGAAGTGGGGAGAGGAGGGATCAGCG BamHI |
| pGBKT7-*VaERF16* D3 |  | CGGAATTCTCTGAAAATAGGGTTGCTCCAAAGC EcoRI | AACTGCAGTCAAAACGCAGCGCTGCATACATGC PstI |
| pGADT7-*VaMYB306* |  | CGGAATTCATGGGAAGACCACCTTGCTGTGATA EcoRI | CGGGATCCTCAGAAAAAATCAGGACTTTCATCT BamHI |
| *VaERF16*-YFPCE |  | CGGGATCCATGTGTGGAGGTGCAATTATATCCG BamHI | GGGGTACCAAACGCAGCGCTGCATACATGCAGA KpnI |
| *VaMYB306*-YFPCE |  | CGGGATCCATGGGAAGACCACCTTGCTGTGATA BamHI | GGGGTACCGAAAAAATCAGGACTTTCATCTAAT KpnI |
| *VaERF16*-YFPNE |  | CGGGATCCATGTGTGGAGGTGCAATTATATCCG BamHI | GGGGTACCAAACGCAGCGCTGCATACATGCAGA KpnI |
| *VaMYB306*-YFPNE |  | CGGGATCCATGGGAAGACCACCTTGCTGTGATA BamHI | GGGGTACCGAAAAAATCAGGACTTTCATCTAAT KpnI |
| *VaERF16*-GFP |  | GGGGTACCATGTGTGGAGGTGCAATTATATCCG KpnI | GCTCTAGAAAACGCAGCGCTGCATACATGCAGA XbaI |
| pEarleyGate201-*VaMYB306* |  | ggggacaagtttgtacaaaaaagcaggctgcATGGGAAGACCACCTTGC | ggggaccactttgtacaagaaagctgggtcGAAAAAATCAGGACTTTC |
| pCB1300-Cluc-*VaERF16* |  | ggggacaagtttgtacaaaaaagcaggctgcATGTGTGGAGGTGCAATT | ggggaccactttgtacaagaaagctgggtcTCAAAACGCAGCGCTGCAT |
| pCB1300-*VaMYB306*-Nluc |  | ggggacaagtttgtacaaaaaagcaggctgcATGGGAAGACCACCTTGC | ggggaccactttgtacaagaaagctgggtcGAAAAAATCAGGACTTTCA |
| pABAi-*ProVaPDF1.2* | XM_002272877 | cttgaattcgagctcggtaccAAAGTAAGTCTTTGAATCATATACTTCTCAAA | agcacatgcctcgaggtcgacCTCTGCACAGCACAGGACATCA |
| pGADT7-*VaERF16* |  | CGGAATTCATGTGTGGAGGTGCAATTATATCCG EcoRI | CGGGATCCTCAAAACGCAGCGCTGCATACATGC BamHI |
| 62-SK-*VaERF16* |  | cgctctagaactagtggatccATGTGTGGAGGTGCAATTATATCC | gataagcttgatatcgaattcAAACGCAGCGCTGCATACA |
| 62-SK-*VaMYB306* |  | cgctctagaactagtggatccATGGGAAGACCACCTTGCTGT | gataagcttgatatcgaattcGAAAAAATCAGGACTTTCATCTAATGA |
| *ProVaPDF1.2*-LUC |  | ctatagggcgaattgggtaccAAAGTAAGTCTTTGAATCATATACTTCTCAAA | aagcttatcgataccgtcgacCTCTGCACAGCACAGGACATCA |
| *VaERF16*-RNAi-sense |  | CCCTCGAGATGTGTGGAGGTGCAATTATATCCG XhoI | GGGGTACCGTTCTTCCGAATCCTCGGAGTGGTG KpnI |
| *VaERF16*-RNAi-antisense |  | GCTCTAGAATGTGTGGAGGTGCAATTATATCCG XbaI | CCATCGATGTTCTTCCGAATCCTCGGAGTGGTG ClaI |
| *VaMYB306*-RNAi-sense |  | CCCTCGAGGATGTGTCCCTAGCATCACAGTCAA XhoI | GGGGTACCCGAGCAATGTTCTCAGTACTGGATG KpnI |
| *VaMYB306*-RNAi-antisense |  | GCTCTAGAGATGTGTCCCTAGCATCACAGTCAA XbaI | CCATCGATCGAGCAATGTTCTCAGTACTGGATG ClaI |
| 35S-*VaERF16* |  | GCTCTAGAATGTGTGGAGGTGCAATTATATCCG XbaI | GGGGTACCTCAAAACGCAGCGCTGCATACATGC KpnI |
| **Primers used for qRT-PCR** | | | |
| qRT-*ERF16* | CBI22960.3 | AGGGTGTGAGAGTGTGGCT | CTTGACTGTGCTGTGGGGT |
| qRT-*AtPDF1.2* | NM_123809 | TCCATCATCACCCTTATCTTCG | GCACTGATTCTTGCATGCATTACTG |
| qRT-*AtLOX3* | NP_564021 | TCTCCGTACAACAAGCGTTGG | GCGTCCGTCTAGCGCATTAAT |
| qRT-*AtPR1* | NP_179068 | GGAGCTACGCAGAACAACTAAGA | CCCACGAGGATCATAGTTGCAACTGA |
| qRT-*AtPR3* | NM_112085 | CTGACCTTGTTGCCAACGAC | ACGTCCACACTCCAATCCAC |
| qRT-*AtPR4* | NP_187123 | TGTCCCGGTAACATCTGCTG | AAGCACTCACGGCTCTCAAA |
| qRT-*AtNPR1* | NP_176610 | CTTGCGGAGAAGACGACACT | TCACCGACGACGATGAGAGA |
| qRT-*MYB306* | XP_002283575 | ACTAATGGCTATCAGACTTGC | CGAATTTCTCATCCACCCT |
| qRT-*PDF1.2* | XM_002272877 | TCCTCCTCCTCATCCTCTTGG | ACAGAGCTTGGTGCAGAAGC |
| qRT-*ERF20* | XP_010650070 | AAACGACTCCCAGGAGATGC | CTTATGCCATGCCTGGTCGA |
| qRT-*AtRBOHD* | NM_124165 | ACGTGCGTCCAAGAAAAACG | CGTAAGAAGGGCTAGCTCCG |
| qRT-*AtRBOHF* | NM_105079 | TCAGAGCCGACGAAACAACA | TCCGAGATCGAATCCGCATG |
| qRT-*B.cinerea Actin* | XM_024697950 | GCTGGTCGTGATTTGACTGAT | GACTGGCGGTTTGGATTTCTT |
| qRT-*AtActin2* | AT3G18780 | CTTGCACCAAGCAGCATGAA | CCGATCCAGACACTGTACTTCCTT |
| qRT-*EF1α* | AT5G60390 | TCCAGCTAAGGGTGCC | GGTGGGTACTCGGAGA |
| qRT-*UBQ5* | AT3G62250 | GACGCTTCATCTCGTCC | CCACAGGTT GCGTTAG |
| qRT-*GAPDH* | XM_002278316.4 | CATTGTGCCAACATCCACGG | GGAGCTCCTTCTCAGCACTG |
| qRT-*EF1-α* | XM_002284888 | AGGAGGCAGCCAACTTCACC | CAAACCCTGCATCACCATTC |
| qRT-*Actin7* | NP_001327851.1 | GATTCTGGTGATGGTGTGAGT | GACAATTTCCCGTTCAGCAGT |
